# (1*R*,2*S*,4*r*)-1,2,4-Tri­phenyl­cyclo­pentane-1,2-diol and (1*R*,2*S*,4*r*)-4-(2-meth­oxy­phen­yl)-1,2-di­phenyl­cyclo­pentane-1,2-diol: application as initiators for ring-opening polymerization of ∊-caprolactone

**DOI:** 10.1107/S2056989019008673

**Published:** 2019-06-21

**Authors:** Pavel D. Komarov, Mikhail E. Minyaev, Andrei V. Churakov, Dmitrii M. Roitershtein, Ilya E. Nifant’ev

**Affiliations:** a A.V. Topchiev Institute of Petrochemical Synthesis, Russian Academy of Sciences, 29 Leninsky prospect, 119991, Moscow, Russian Federation; b N.S. Kurnakov Institute of General and Inorganic Chemistry, Russian Academy of Sciences, 31 Leninsky Prospect, Moscow, 119991, Russian Federation; c N.D. Zelinsky Institute of Organic Chemistry, Russian Academy of Sciences, 47 Leninsky Prospect, Moscow, 119991, Russian Federation; d Chemistry Department, M.V. Lomonosov Moscow State University, 1 Leninskie Gory Str., Building 3, Moscow 119991, Russian Federation

**Keywords:** cyclo­pentane-1,2-diol, crystal structure, hydrogen bonding, ring-opening polymerization, caprolactone

## Abstract

Reductive cyclization of 1,3,5-triphenyl- and 3-(2-meth­oxy­phen­yl)-1,5-di­phenyl­pentane-1,5-diones by zinc in acetic acid medium leads to the formation of 1,2,4-tri­phenyl­cyclo­pentane-1,2-diol [1,2,4-Ph_3_C_5_H_5_-1,2-(OH)_2_, C_23_H_22_O_2_, (**I**)] and 4-(2-meth­oxy­phen­yl)-1,2-di­phenyl­cyclo­pentane-1,2-diol [4-(2-MeOC_6_H_4_)-1,2-Ph_2_C_5_H_5_-1,2-(OH)_2_, C_24_H_24_O_3_, (**II**)]. Their single crystals have been obtained by crystallization from a THF/hexane solvent mixture. Diols (**I**) and (**II**) crystallize in ortho­rhom­bic (*Pbca*) and triclinic (*P* ) space groups, respectively, at 150 K. Their asymmetric units comprise one [in the case of (**I**)] and three [in the case of (**II**)] crystallographically independent mol­ecules of the achiral (1*R*,2*S*,4*r*)-diol isomer. Each hydroxyl group is involved in one intra­molecular and one inter­molecular O—H⋯O hydrogen bond, forming one-dimensional chains. Compounds (**I**) and (**II**) have been used successfully as precatalyst activators for the ring-opening polymerization of ∊-caprolactone.

## Abstract 2

Achiral (1*R*,2*S*,4*r*)-1,2,4-tri­phenyl­cyclo­pentane-1,2-diol and (1*R*,2*S*,4*r*)-4-(2-meth­oxy­phen­yl)-1,2-di­phenyl­cyclo­pentane-1,2-diol form one-dimensional chains *via* O—H⋯O hydrogen bonding in their crystals. The diols may serve as precatalyst activators for ring-opening polymerization of cyclic esters.

## Chemical context   

1,2,4-Tri­aryl­cyclo­pentane-1,2-diols are useful synthetic precursors for obtaining 1,2,4-tri­aryl­cyclo­penta-1,3-dienes (Hirsch & Bailey, 1978 ▸; Yang *et al.*, 2012 ▸; Zhang *et al.*, 2013 ▸; Ye *et al.*, 2016 ▸, 2017 ▸). The latter compounds are currently of inter­est because of their intrinsic luminescent properties due to aggregation-induced emission enhancement (Yang *et al.*, 2012 ▸; Zhang, Ye *et al.*, 2013 ▸; Ye *et al.*, 2016 ▸, 2017 ▸). Certain 4-aryl-1,2-di­phenyl­cyclo­penta-1,3-dienes are promising cand­i­dates for the fabrication of OLED devices (Ye *et al.*, 2017 ▸). However, most tri­aryl­cyclo­penta­dienes are mainly used for the synthesis of the corresponding organometallic cyclo­penta­dienyl complexes. Up to date, the number of known tri­phenyl­cyclo­penta­dienyl complexes of *d*- (Davies *et al.*, 2000 ▸; Deck *et al.*, 2006 ▸; Thornberry *et al.*, 2000 ▸, 2004 ▸; Wu *et al.*, 2007 ▸; Xu *et al.*, 2006 ▸, 2007 ▸; Zhang *et al.*, 2000 ▸; Zhang *et al.*, 2003 ▸) and *f*-block metals (Visseaux *et al.*, 2008 ▸; Minyaev *et al.*, 2016 ▸; Roitershtein *et al.*, 2012 ▸, 2018 ▸) is rather limited, and they are still insufficiently studied. Various polyphenyl­cyclo­penta­dienyl Tb complexes, including 1,2,4-tri­phenyl­cyclo­penta­dienyl ones, display promising photophysical properties because of the presence of such a ligand, which serves as a π-type antenna for luminescence sensitization of lanthanides (Roitershtein *et al.*, 2018 ▸). Organometallic derivatives of *d*- and *f*-block metals with various tri­phenyl­cyclo­penta­dienyl ligands may also be used in the catalytic polymerization of olefins (Thornberry *et al.*, 2004 ▸; Visseaux *et al.*, 2008 ▸; Minyaev *et al.*, 2016 ▸; Xu *et al.*, 2006 ▸, 2007 ▸; Zhang *et al.*, 2000 ▸; Zhang *et al.*, 2003 ▸).
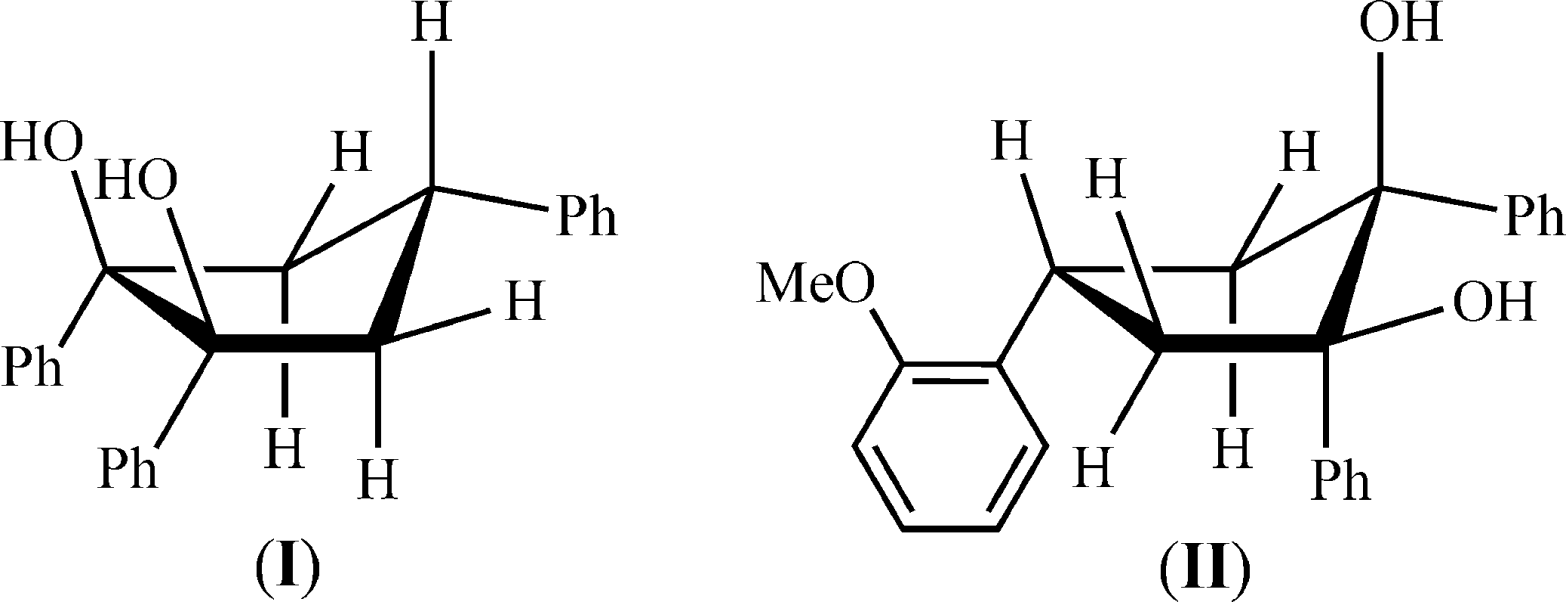


**Figure 1:**
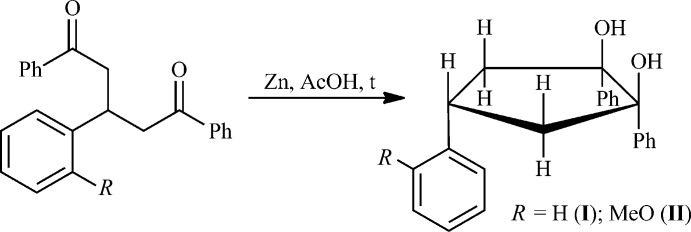
Synthesis of 1,2,4-tri­phenyl­cyclo­pentane-1,2-diol (**I**) and 4-(2-meth­oxy­phen­yl)-1,2-di­phenyl­cyclo­pentane-1,2-diol (**II**).

1,2-Diphenyl-4-aryl­cyclo­pentane-1,2-diols can be readily synthesized by the reductive cyclization of 1,5-diphenyl-3-aryl­pentane-1,5-diones with zinc in an acetic acid medium (Fig. 1 ▸; aryl = Ph, 2-MeOC_6_H_4_). The corresponding diones are formed by condensation of aceto­phenone with benzaldehyde/2-meth­oxy­benzaldehyde under basic conditions (Hirsch & Bailey, 1978 ▸; Minyaev *et al.*, 2015 ▸). The presence of only one isomer (see §2) has been detected by ^1^H NMR studies in the samples of all isolated crystalline diols from repeated syntheses. However, examination of the reaction mixtures has allowed us to suppose that another minor isomer of (**I**) may sometimes be present (up to 20%), but it does not crystallize under the conditions used here.

**Figure 2:**
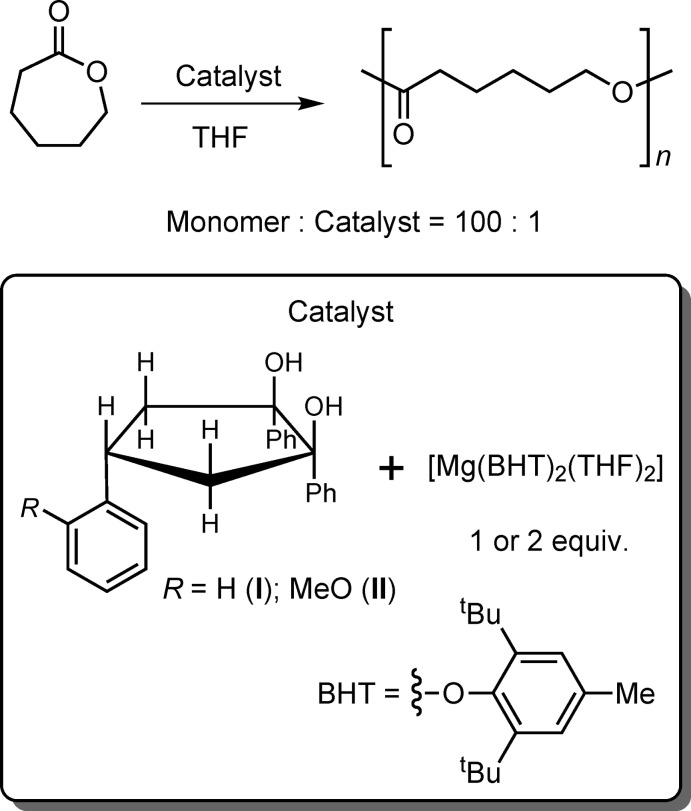
Ring-opening polymerization of ∊-caprolactone using [Mg(BHT)_2_(THF)_2_] and either (**I**) or (**II**).

It is known that complexes [Mg(BHT)(O*R*)(THF)_*n*_]_2_ (*n* = 0, 1; BHT = *O*-2,6-^*t*^Bu_2_-4-MeC_6_H_2_ or the anion of butyl­ated hy­droxy­toluene) are active in ring-opening polymerization (ROP) of cyclic esters (Nifant’ev *et al.*, 2016 ▸, 2017 ▸), whereas Mg(BHT)_2_(THF)_2_ is catalytically inactive, but displays relatively high catalytic activity upon activation by a primary alcohol (see, for example, Chen *et al.*, 2012 ▸). The ROP of ∊-caprolactone (∊-CL) to poly(∊-caprolactone) (PCL) can be carried out on the precatalyst Mg(BHT)_2_(THF)_2_ activated even by various bulky alcohols (Minyaev *et al.*, 2018 ▸). We have tested the obtained diols (**I**) and (**II**) as activators of the Mg(BHT)_2_(THF)_2_ precatalyst for polymerization of ∊-CL (Fig. 2 ▸, Table 1 ▸). In all cases, the qu­anti­tative conversion of ∊-CL to PCL was observed by ^1^H NMR spectroscopy.

In the case of the ratio [diol]/[Mg(BHT)_2_] = 1:1 (entries 1 and 3, Table 1 ▸), the polymerization degree (the number of polymerized monomer units, *P_n_*) found by ^1^H NMR spectroscopy and by size-exclusion chromatography (SEC) are very close to the calculated value (*P_n calcd._ =* 100). However, when the ratio [diol]/[Mg(BHT)_2_] = 1:2, and two chains are growing at one diol, the *P_n_* values (entries 2 and 4) are somewhat higher than expected (*P_n calcd._* = 50), which might be explained by a longer reaction time of the second [Mg(BHT)_2_(THF)_2_] mol­ecule with the same initiator mol­ecule with respect to the time of polymer-chain propagation. This is also supported by larger polydispersity index (*Đ*) values (compare entries 2 and 4 with entries 1 and 3), pointing to unequal growth of the two chains.

Therefore, catalytic tests have shown that systems based on [Mg(BHT)_2_(THF)_2_] and (**I**) or (**II**) are capable of catalysing ROP of ∊-CL, providing 100% monomer conversion. When using the diol/Mg(BHT)_2_ ratio equal to 1:1, the ROP can be carried out in a more controlled manner.

## Structural commentary   

**Figure 3:**
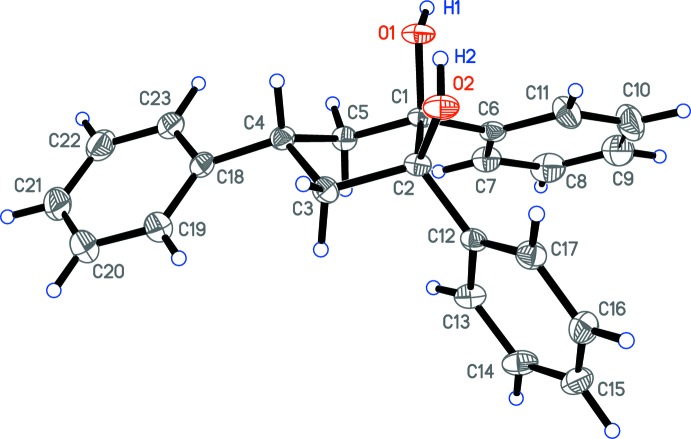
The structure of (1*R*,2*S*,4*r*)-1,2,4-tri­phenyl­cyclo­pentane-1,2-diol, (**I**). Displacement ellipsoids for non-H atoms are drawn at the 50% probability level. Only hy­droxy H atoms are labelled for clarity. The intra­molecular hydrogen bonding is not shown.

**Figure 4:**
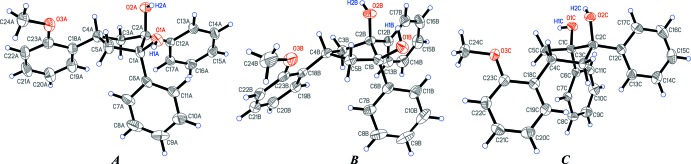
The structure of the three crystallographically independent mol­ecules (***A***, ***B***, ***C***) of (1*R*,2*S*,4*r*)-4-(2-meth­oxy­phen­yl)-1,2-di­phenyl­cyclo­pentane-1,2-diol, (**II**). Displacement ellipsoids for non-H atoms are drawn at the 50% probability level. Only hy­droxy H atoms are labelled for clarity. The intra­molecular hydrogen bonding is not shown.

Compounds (**I**) and (**II**) crystallize in the ortho­rhom­bic *Pbca* and triclinic *P* 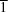 space groups, respectively. The asymmetric units of (**I**) and (**II**) contain one and three diol mol­ecules, respectively, exhibiting an achiral configuration (1*R*,2*S*,4*r*) with all three phenyl groups being on one side of the cyclo­pentane ring (Figs. 3 ▸ and 4 ▸). However, the envelope conformations of (**I**) and (**II**) differ, which might be caused by crystal-packing effects. Thus, atoms C1, C2, C3 and C5 in (**I**) lie nearly in one plane but atom C4 deviates by 0.6727 (19) Å from the plane (see Scheme and Fig. 3 ▸). All three crystallographically independent mol­ecules in (**II**) (***A***, ***B*** and ***C***; Fig. 4 ▸) have very similar envelope conformations (with the exception of the positions of the hy­droxy H atoms), with atom C2 being out of the plane formed by atoms C1, C3, C4 and C5 by 0.644 (3), 0.666 (3) and 0.633 (3) Å in (**II** ***A***), (**II** ***B***) and (**II** ***C***), respectively (see Scheme[Chem scheme1] and Fig. 4 ▸). A conformation which is very similar to those of mol­ecules (**II** ***A***), (**II** ***B***) and (**II** ***C***) has been found earlier for (1*R*,2*S*)-1,2-di­phenyl­cyclo­pentane-1,2-diol, having the Cambridge Structural Database (Version 5.40; Groom *et al.*, 2016 ▸) refcode ZIWVEG (Choi *et al.*, 1995 ▸). All C—C and C—O bond distances in (**I**) and (**II**) fall into regular ranges and can be found in the supporting information.

**Figure 5:**
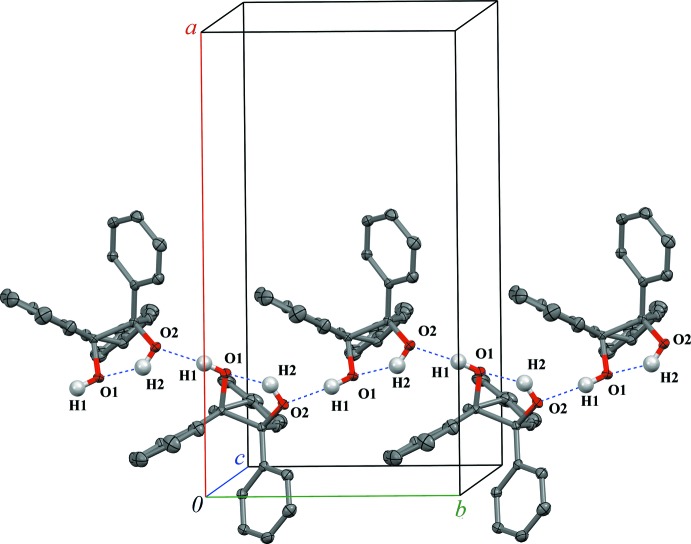
The one-dimensional chains formed by hydrogen bonding between mol­ecules of (1*R*,2*S*,4*r*)-1,2,4-tri­phenyl­cyclo­pentane-1,2-diol (**I**) parallel to the *b* axis. Displacement ellipsoids are drawn at the 50% probability level. Non-hy­droxy H atoms are not shown.

Diols (**I**) and (**II**) each form one intra­molecular O—H⋯O hydrogen bond: O2—H2⋯O1 for (**I**), O2*A*—H2*A*⋯O1*A* for (**II** ***A***), O1*B*—H1*B*⋯O2*B* for (**II** ***B***) and O2*C*—H2*C*⋯O1*C* for (**II** ***C***) (Figs. 5 ▸, 6 ▸). The corresponding O—H⋯O bond angles range from 117 (2)° in (**II** ***B***) to 131.0 (19)° in (**I**) (Tables 2 ▸ and 3 ▸).

## Supra­molecular features   

Regardless of some structural differences, diols (**I**) and (**II**) form similar 1D chains in their crystals *via* inter­molecular O—H⋯O hydrogen bonding [O1—H1⋯O2^i^ for (**I**), symmetry code: (i) −*x* + 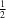, *y* − 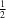, *z*; and O2*B*—H2*B*⋯O2*A*, O1*C*—H1*C*⋯O1*B*, O1*A*—H1*A*⋯O2*C* ^ii^ for (**II**), symmetry code: (ii) *x* − 1, *y* − 1, *z*]. The inter­molecular O—H⋯O bond angles lie in the expected range of 160 (3) to 173.2 (19)°. The chains are oriented along the *b*-axis direction in (**I**) and approximately along the *ab* diagonal in (**II**). It might be also mentioned that for both the inter- and intra­molecular hydrogen bonds, the O⋯O and consequently O—H⋯O distances are slightly elongated in (**II**) compared to (**I**), likely as a result of crystal-packing effects.

## Synthesis and crystallization   

### General remarks   

The starting compounds 1,3,5-tri­phenyl­pentane-1,5-dione and 3-(2-meth­oxy­phen­yl)-1,5-di­phenyl­pentane-1,5-dione were obtained in high yields by the previously described procedure (Hirsch & Bailey, 1978 ▸) with certain minor modifications (Minyaev *et al.*, 2015 ▸) to decrease formation of side products. They were recrystallized from hot ethanol or iso­propanol followed by vacuum drying. The complex Mg(BHT)_2_(THF)_2_ was prepared as described earlier (Nifant’ev *et al.*, 2017 ▸). All polymerization tests and the synthesis of Mg(BHT)_2_(THF)_2_ were performed under a purified argon atmosphere in a dry box in absolute solvent media. Tetra­hydro­furan was pre-dried over NaOH and distilled from potassium/benzo­phenone ketyl. Hexane was distilled from an Na/K alloy. Toluene was distilled from sodium/benzo­phenone ketyl. ∊-Caprolactone (∊-CL) was distilled from CaH_2_ under reduced pressure of argon. CDCl_3_ (Cambridge Isotope Laboratories, Inc., D 99.8%) was used as purchased. The NMR spectra were recorded on Bruker AV400 and AV600 spectrometers at 300 K; chemical shifts are reported in ppm relative to the solvent residual peak. The SEC analysis of polymer samples was performed at 323 K using an Agilent PL-GPC 220 gel permeation chromatograph equipped with a PLgel column, with DMF as eluent (1 ml min^−1^) and poly(ethyl­ene oxide) standards.

### Synthesis and crystallization of (I)   

(1*R*,2*S*,4*r*)-1,2,4-Tri­phenyl­cyclo­pentane-1,2-diol, (**I**), was prepared as described previously (Hirsch & Bailey, 1978 ▸) in a yield of 78%, m.p. = 415–417K. ^1^H NMR (400 MHz, CDCl_3_): δ = 2.61 (2H, *dd*, –C***H_2_***–, ^2^ *J*_HH_ = 13.9 Hz, ^3^ *J*_HH_ = 8.8 Hz), 2.84 (2H, *dd*, –C***H_2_***–, ^2^ *J*_HH_ = 13.9 Hz, ^3^ *J*_HH_ = 10.1 Hz), 3.58 (2H, *s*, >CPh—O***H***), 4.11 (1H, *quintet*, >CPh***H***), 6.97–7.12 (10H, *m*), 7.15–7.36 (2H, *m*), 7.43 (3H, *d*, ^3^ *J*_HH_ = 4.0 Hz).

A small portion of (**I**) was dissolved in a warm mixture of THF/hexane (1:10 *v*/*v*) to provide a saturated solution. Single crystals formed in two weeks.

### Synthesis and crystallization of (II)   

(1*R*,2*S*,4*r*)-4-(2-Meth­oxy­phen­yl)-1,2-di­phenyl­cyclo­pentane-1,2-diol, (**II**), was prepared analogously to (**I**) but with some minor modifications. Zinc powder (20.00 g, 306 mmol) was added by small portions over 5 h to a vigorously stirred solution of 1,5-diphenyl-3-(2-meth­oxy­phen­yl)pentane-1,5-dione (27.43 g, 76.5 mmol) in 900 ml of glacial acetic acid at 363 K. The formed hot mixture was filtered. The resulting solution was cooled to room temperature and poured into 5000 ml of water. The formed yellowish precipitate was collected, washed with water (2 × 100 ml) and dried under vacuum. The solid was recrystallized from a hot mixture of petroleum ether (boiling temperature range of 343–373 K) and toluene (400 ml, 3:1 *v*/*v*). The white microcrystals were dried under dynamic vacuum. The yield was 17.42 g (48.3 mmol, 63.2%), (m.p. = 384–387 K. ^1^H NMR (600 MHz, CDCl_3_): δ = 2.56 (2H, *dd*, –C***H_2_***–, ^2^ *J*_HH_ = 14.3 Hz, ^3^ *J*_HH_ = 8.4 Hz), 2.81 (2H, *dd*, –C***H_2_***–, ^2^ *J*_HH_ = 14.3 Hz, ^3^ *J*_HH_ = 10.3 Hz), 3.46 (2H, *s*, >CPh—O***H***), 3.90 (3H, *s*, –OC***H_3_***), 4.27 [1H, *quintet*, –C(C_6_H_4_OMe)***H***], 6.96 (1H, *d*, ^3^ *J*_HH_ = 8.1 Hz), 7.02–7.07 (7H, *m*), 7.09–7.13 (4H, *m*), 7.31 (1H, *t*), 7.45 (1H, *d*, ^3^ *J*_HH_ = 7.3 Hz). ^13^C{^1^H} NMR (150.9 MHz, CDCl_3_): δ = 34.07, 44.21, 55.40, 85.58, 110.67, 120.52, 126.34, 126.47, 126.82, 127.34, 127.48, 132.17, 143.51, 158.26.

Single crystals of (**II**), suitable for X-ray diffraction analysis, were grown from a THF/hexane mixture (1:10 *v*/*v*) over two weeks.

### Polymerization procedure   

In a typical polymerization experiment, a solution of 0.1 mmol of a diol [33 mg of (**I**) or 36 mg of (**II**)] in 1 ml of THF was added to a stirred solution of Mg(BHT)_2_(THF)_2_ (0.1 mmol, 61 mg or 0.2 mmol, 121 mg) in 1 ml of THF. The resulting solution was stirred for 20 min. A solution of ∊-CL (1.14 g, 10 mmol) in 1 ml of THF was then added at once to the formed catalyst solution. The solution was stirred for 30 min and then a sample was taken to determine conversion of the monomer by ^1^H NMR spectroscopy. A 100% conversion was established in all cases based on the absence of a resonance signal at 4.22 ppm (∊-CL) and the presence of a signal at 4.05 ppm (PCL), both corresponding to the –C***H_2_***O(CO)– fragment. The remaining viscous solution was poured into methanol (50 ml) containing a drop of acetic acid. The resulting precipitate was separated by centrifugation, washed with methanol (3 × 25 ml) and hexane (2 × 10 ml) and dried under vacuum. Polymer samples were further studied by SEC and ^1^H NMR analysis. The degree of polymerization was determined by integration of a PCL terminal group signal at 3.63 ppm (–CH_2_—C***H_2_***—OH).

## Refinement   

Crystal data, data collection and structure refinement details are summarized in Table 4 ▸. The positions of all hydrogen atoms in (**I**) and the hy­droxy H atoms in (**II**) were found from the difference maps. These H atoms were refined independently with individual isotropic displacement parameters. The other H atoms in (**II**) were positioned geometrically (C—H = 0.95 Å for aromatic, 0.98 Å for methyl, 0.99 Å for methyl­ene and 1.00 Å for methine H atoms) and refined as riding atoms with relative isotropic displacement parameters *U*_iso_(H)= 1.5*U*_eq_(C) for methyl H atoms and 1.2*U*_eq_(C) otherwise. A rotating group model was applied for methyl groups. For (**II**), reflections 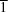
10 and 221 were affected by the beam stop and were omitted from the refinement. The extinction correction in *SHELXL* was used for (**II**) (Sheldrick, 2015 ▸).

## Supplementary Material

Crystal structure: contains datablock(s) I, II, New_Global_Publ_Block. DOI: 10.1107/S2056989019008673/fy2139sup1.cif

Structure factors: contains datablock(s) I. DOI: 10.1107/S2056989019008673/fy2139Isup2.hkl

Structure factors: contains datablock(s) II. DOI: 10.1107/S2056989019008673/fy2139IIsup3.hkl

Supporting information file. DOI: 10.1107/S2056989019008673/fy2139Isup4.cml

Supporting information file. DOI: 10.1107/S2056989019008673/fy2139IIsup5.cml

CCDC references: 1929065, 1929064

Additional supporting information:  crystallographic information; 3D view; checkCIF report

## Figures and Tables

**Figure 6:**
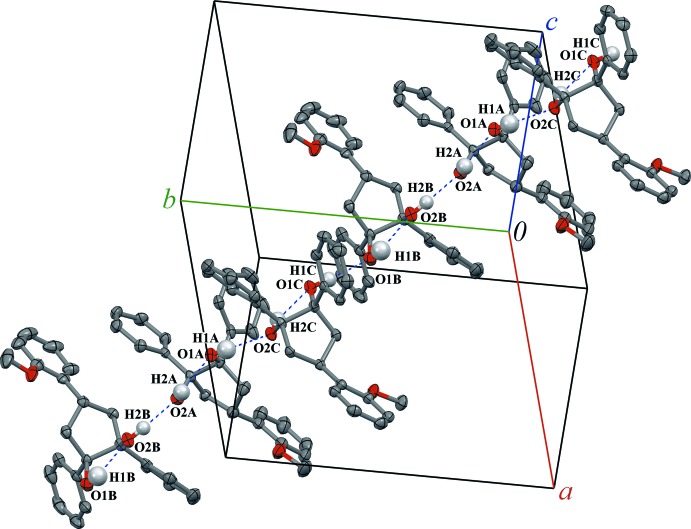
The one-dimensional chains of (1*R*,2*S*,4*r*)-4-(2-meth­oxy­phen­yl)-1,2-di­phenyl­cyclo­pentane-1,2-diol mol­ecules (**II**) along the ***ab*** direction. Displacement ellipsoids are drawn at the 50% probability level. Non-hy­droxy H atoms are not shown.

**Table 1:** Polymerization of ∊-CL *M_n_* is the number-average molar mass; *Đ* is the polydispersity index defined as *Đ*=*M_w_*/*M_n_*, where *M_w_* is the weight-average molar mass; *P_n_* is the polymerization degree. Conditions: [∊-CL] = 2.5 *M*; THF; [∊-CL]/[diol]/[Mg(BHT)_2_] = 100:1:1 or 2; 300 K, 30 min.

Entry	Diol	Equiv. of Mg(BHT)_2_	*M_n_* ×10^3*a*^	*Ð^*a*^*	*P_n_^*a*^*	*M_n_* ×10^3*b*^	*P_n_^*b*^*
1	(**I**)	1	11.4	1.42	97	12.0	102
2	(**I**)	2	9.0	1.84	77	7.6	65
3	(**II**)	1	12.4	1.39	106	12.6	107
4	(**II**)	2	8.9	1.85	76	7.2	62

Notes: (*a*) Found by size-exclusion chromatography (SEC) measurements. (*b*) Determined by ^1^H NMR studies. *M_n_* and *P_n_* were calculated based on the end-group analysis.

**Table 2:** Hydrogen-bond geometry (Å, °) for **I** [Chem scheme1]

*D*—H⋯*A*	*D*—H	H⋯*A*	*D*⋯*A*	*D*—H⋯*A*
O1—H1⋯O2^i^	0.87 (2)	1.89 (2)	2.7509 (13)	173.2 (19)
O2—H2⋯O1	0.86 (2)	1.80 (2)	2.4510 (14)	131.0 (19)

Symmetry code: (i) 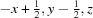.

**Table 3:** Hydrogen-bond geometry (Å, °) for (**II**)

*D*—H⋯*A*	*D*—H	H⋯*A*	*D*⋯*A*	*D*—H⋯*A*
O1*A*—H1*A*⋯O2*C* ^ii^	0.85 (3)	2.08 (3)	2.8931 (19)	160 (3)
O2*A*—H2*A*⋯O1*A*	0.88 (3)	2.04 (3)	2.605 (2)	121 (2)
O1*B*—H1*B*⋯O2*B*	0.90 (3)	2.05 (3)	2.590 (2)	117 (2)
O2*B*—H2*B*⋯O2*A*	0.83 (2)	1.98 (2)	2.802 (2)	170 (2)
O1*C*—H1*C*⋯O1*B*	0.88 (3)	1.96 (3)	2.833 (2)	171 (2)
O2*C*—H2*C*⋯O1*C*	0.85 (3)	2.00 (3)	2.587 (2)	125 (2)

Symmetry code: (ii) 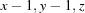.

**Table 4:** Experimental details

	(**I**)	(**II**)
Crystal data		
Chemical formula	C_23_H_22_O_2_	C_24_H_24_O_3_
*M*_r_	330.40	360.43
Crystal system, space group	Orthorhombic, *P* *b* *c* *a*	Triclinic, *P* 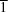
Temperature (K)	150	150
*a*, *b*, *c* (Å)	16.9915 (6), 9.3183 (3), 22.0129 (7)	11.4136 (6), 14.0145 (7), 19.0339 (10)
α, β, γ (°)	90, 90, 90	92.3394 (18), 101.5461 (17), 105.0129 (19)
*V* (Å^3^)	3485.3 (2)	2867.3 (3)
*Z*	8	6
Radiation type	Mo *K*α	Mo *K*α
μ (mm^−1^)	0.08	0.08
Crystal size (mm)	0.40 × 0.35 × 0.20	0.50 × 0.20 × 0.10

Data collection		
Diffractometer	Bruker SMART APEXII	Bruker SMART APEXII
Absorption correction	Multi-scan (*SADABS*; Krause *et al.*, 2015 ▸)	Multi-scan (*SADABS*; Krause *et al.*, 2015 ▸)
*T*_min_, *T*_max_	0.869, 0.928	0.856, 0.928
No. of measured, independent and observed [*I* > 2σ(*I*)] reflections	39896, 4625, 4025	31335, 11202, 8043
*R*_int_	0.041	0.039
(sin θ/λ)_max_ (Å^−1^)	0.682	0.617

Refinement		
*R*[*F* ^2^ > 2σ(*F* ^2^)], *wR*(*F* ^2^), *S*	0.055, 0.136, 1.11	0.051, 0.136, 1.04
No. of reflections	4625	11202
No. of parameters	314	758
H-atom treatment	All H-atom parameters refined	H atoms treated by a mixture of independent and constrained refinement
Δρ_max_, Δρ_min_ (e Å^−3^)	0.40, −0.23	0.36, −0.24

Computer programs: *APEX3* and *SAINT* (Bruker, 2018 ▸), *SHELXS* and *SHELXTL* (Sheldrick, 2008 ▸), *SHELXL2017* (Sheldrick, 2015 ▸), *publCIF* (Westrip, 2010 ▸) and *Mercury* (Macrae *et al.*,2006 ▸).

## supplementary crystallographic information

### (1R,2S,4r)-1,2,4-Triphenylcyclopentane-1,2-diol (I) Crystal data

**Table:**

C_23_H_22_O_2_	*D*_x_ = 1.259 Mg m^−^^3^
*M**_r_* = 330.40	Mo *K*α radiation, λ = 0.71073 Å
Orthorhombic, *P**b**c**a*	Cell parameters from 9996 reflections
*a* = 16.9915 (6) Å	θ = 3.0–30.4°
*b* = 9.3183 (3) Å	µ = 0.08 mm^−^^1^
*c* = 22.0129 (7) Å	*T* = 150 K
*V* = 3485.3 (2) Å^3^	Prism, colourless
*Z* = 8	0.40 × 0.35 × 0.20 mm
*F*(000) = 1408	

### (1R,2S,4r)-1,2,4-Triphenylcyclopentane-1,2-diol (I) Data collection

**Table:**

Bruker SMART APEXII diffractometer	4625 independent reflections
Radiation source: fine-focus sealed tube	4025 reflections with *I* > 2σ(*I*)
Graphite monochromator	*R*_int_ = 0.041
ω scans	θ_max_ = 29.0°, θ_min_ = 2.2°
Absorption correction: multi-scan (SADABS; Krause *et al.*, 2015)	*h* = −22→23
*T*_min_ = 0.869, *T*_max_ = 0.928	*k* = −12→12
39896 measured reflections	*l* = −30→29

### (1R,2S,4r)-1,2,4-Triphenylcyclopentane-1,2-diol (I) Refinement

**Table:**

Refinement on *F*^2^	Primary atom site location: structure-invariant direct methods
Least-squares matrix: full	Secondary atom site location: difference Fourier map
*R*[*F*^2^ > 2σ(*F*^2^)] = 0.055	Hydrogen site location: difference Fourier map
*wR*(*F*^2^) = 0.136	All H-atom parameters refined
*S* = 1.11	*w* = 1/[σ^2^(*F*_o_^2^) + (0.0649*P*)^2^ + 1.4502*P*] where *P* = (*F*_o_^2^ + 2*F*_c_^2^)/3
4625 reflections	(Δ/σ)_max_ < 0.001
314 parameters	Δρ_max_ = 0.40 e Å^−^^3^
0 restraints	Δρ_min_ = −0.23 e Å^−^^3^

### (1R,2S,4r)-1,2,4-Triphenylcyclopentane-1,2-diol (I) Special details

**Table:**

Geometry. All esds (except the esd in the dihedral angle between two l.s. planes) are estimated using the full covariance matrix. The cell esds are taken into account individually in the estimation of esds in distances, angles and torsion angles; correlations between esds in cell parameters are only used when they are defined by crystal symmetry. An approximate (isotropic) treatment of cell esds is used for estimating esds involving l.s. planes.
Refinement. Refinement of F^2^ against ALL reflections. The weighted R-factor wR and goodness of fit S are based on F^2^, conventional R-factors R are based on F, with F set to zero for negative F^2^. The threshold expression of F^2^ > 2sigma(F^2^) is used only for calculating R-factors(gt) etc. and is not relevant to the choice of reflections for refinement. R-factors based on F^2^ are statistically about twice as large as those based on F, and R- factors based on ALL data will be even larger.

### (1R,2S,4r)-1,2,4-Triphenylcyclopentane-1,2-diol (I) Fractional atomic coordinates and isotropic or equivalent isotropic displacement parameters (Å^2^)

**Table:**

	*x*	*y*	*z*	*U*_iso_*/*U*_eq_	
O1	0.24602 (5)	0.55215 (10)	0.18770 (4)	0.0209 (2)	
H1	0.2241 (12)	0.469 (2)	0.1826 (9)	0.037 (5)*	
O2	0.31200 (6)	0.78090 (10)	0.16702 (5)	0.0211 (2)	
H2	0.2699 (13)	0.731 (2)	0.1629 (9)	0.040 (5)*	
C1	0.32785 (7)	0.53090 (13)	0.20268 (6)	0.0154 (2)	
C2	0.36409 (7)	0.69000 (13)	0.20090 (6)	0.0156 (2)	
C3	0.36604 (8)	0.73633 (14)	0.26866 (6)	0.0197 (3)	
H3A	0.4214 (10)	0.7274 (18)	0.2843 (8)	0.026 (4)*	
H3B	0.3500 (10)	0.8380 (19)	0.2727 (7)	0.023 (4)*	
C4	0.31240 (7)	0.63121 (14)	0.30256 (6)	0.0182 (3)	
H4	0.2562 (10)	0.6555 (17)	0.2932 (7)	0.019 (4)*	
C5	0.33277 (8)	0.49036 (13)	0.26981 (6)	0.0185 (3)	
H5A	0.2978 (10)	0.4132 (19)	0.2798 (8)	0.026 (4)*	
H5B	0.3860 (9)	0.4584 (17)	0.2823 (7)	0.018 (4)*	
C6	0.36313 (7)	0.42551 (13)	0.15783 (6)	0.0178 (3)	
C7	0.39703 (8)	0.29720 (14)	0.17635 (7)	0.0233 (3)	
H7	0.3998 (10)	0.2754 (19)	0.2195 (9)	0.030 (5)*	
C8	0.42672 (9)	0.20104 (17)	0.13381 (8)	0.0327 (4)	
H8	0.4512 (13)	0.112 (2)	0.1483 (9)	0.045 (6)*	
C9	0.42272 (10)	0.23193 (19)	0.07249 (8)	0.0371 (4)	
H9	0.4433 (12)	0.164 (2)	0.0424 (10)	0.049 (6)*	
C10	0.38891 (11)	0.3595 (2)	0.05359 (7)	0.0386 (4)	
H10	0.3878 (13)	0.384 (2)	0.0106 (10)	0.050 (6)*	
C11	0.35920 (9)	0.45546 (17)	0.09590 (7)	0.0285 (3)	
H11	0.3358 (11)	0.546 (2)	0.0819 (8)	0.034 (5)*	
C12	0.44573 (7)	0.70012 (13)	0.17266 (6)	0.0163 (2)	
C13	0.50697 (8)	0.61131 (14)	0.19234 (6)	0.0209 (3)	
H13	0.4978 (11)	0.541 (2)	0.2244 (8)	0.032 (5)*	
C14	0.58129 (8)	0.62098 (15)	0.16632 (7)	0.0249 (3)	
H14	0.6240 (11)	0.556 (2)	0.1803 (8)	0.033 (5)*	
C15	0.59666 (8)	0.72139 (16)	0.12135 (7)	0.0259 (3)	
H15	0.6492 (11)	0.730 (2)	0.1042 (8)	0.030 (5)*	
C16	0.53730 (9)	0.81218 (16)	0.10270 (6)	0.0266 (3)	
H16	0.5474 (11)	0.887 (2)	0.0723 (9)	0.037 (5)*	
C17	0.46217 (8)	0.80077 (15)	0.12783 (6)	0.0219 (3)	
H17	0.4216 (11)	0.860 (2)	0.1137 (8)	0.034 (5)*	
C18	0.32029 (7)	0.62406 (15)	0.37101 (6)	0.0209 (3)	
C19	0.36629 (9)	0.71880 (17)	0.40436 (7)	0.0283 (3)	
H19	0.3956 (11)	0.791 (2)	0.3842 (8)	0.028 (4)*	
C20	0.36941 (10)	0.7090 (2)	0.46775 (8)	0.0374 (4)	
H20	0.4009 (13)	0.777 (2)	0.4892 (10)	0.047 (6)*	
C21	0.32644 (10)	0.6053 (2)	0.49798 (7)	0.0384 (4)	
H21	0.3280 (12)	0.602 (2)	0.5425 (10)	0.044 (5)*	
C22	0.28124 (9)	0.5092 (2)	0.46516 (7)	0.0333 (4)	
H22	0.2512 (12)	0.437 (2)	0.4862 (9)	0.040 (5)*	
C23	0.27780 (8)	0.51904 (17)	0.40237 (6)	0.0255 (3)	
H23	0.2437 (11)	0.4513 (19)	0.3798 (8)	0.029 (4)*	

### (1R,2S,4r)-1,2,4-Triphenylcyclopentane-1,2-diol (I) Atomic displacement parameters (Å^2^)

**Table:**

	*U*^11^	*U*^22^	*U*^33^	*U*^12^	*U*^13^	*U*^23^
O1	0.0128 (4)	0.0168 (4)	0.0331 (5)	−0.0001 (3)	−0.0051 (4)	−0.0042 (4)
O2	0.0159 (4)	0.0154 (4)	0.0319 (5)	0.0033 (3)	−0.0044 (4)	0.0023 (4)
C1	0.0123 (5)	0.0119 (5)	0.0220 (6)	−0.0001 (4)	−0.0030 (4)	−0.0014 (4)
C2	0.0147 (5)	0.0121 (5)	0.0202 (6)	0.0009 (4)	−0.0023 (4)	−0.0011 (4)
C3	0.0210 (6)	0.0169 (6)	0.0211 (6)	−0.0026 (5)	−0.0004 (5)	−0.0044 (5)
C4	0.0155 (5)	0.0187 (6)	0.0205 (6)	0.0014 (4)	0.0001 (5)	−0.0021 (5)
C5	0.0196 (6)	0.0151 (5)	0.0207 (6)	−0.0005 (5)	−0.0005 (5)	−0.0006 (5)
C6	0.0139 (5)	0.0164 (6)	0.0232 (6)	−0.0017 (4)	−0.0018 (4)	−0.0052 (5)
C7	0.0215 (6)	0.0177 (6)	0.0307 (7)	0.0018 (5)	−0.0010 (5)	−0.0026 (5)
C8	0.0287 (7)	0.0225 (7)	0.0470 (9)	0.0059 (6)	−0.0001 (7)	−0.0099 (6)
C9	0.0317 (8)	0.0394 (9)	0.0402 (9)	0.0042 (7)	0.0004 (7)	−0.0231 (7)
C10	0.0436 (9)	0.0476 (10)	0.0245 (7)	0.0074 (8)	−0.0038 (7)	−0.0133 (7)
C11	0.0334 (8)	0.0286 (7)	0.0236 (7)	0.0053 (6)	−0.0064 (6)	−0.0052 (6)
C12	0.0158 (5)	0.0136 (5)	0.0194 (5)	−0.0012 (4)	−0.0023 (4)	−0.0032 (4)
C13	0.0174 (6)	0.0165 (6)	0.0288 (7)	−0.0004 (5)	−0.0020 (5)	0.0025 (5)
C14	0.0167 (6)	0.0195 (6)	0.0385 (8)	0.0017 (5)	−0.0018 (5)	−0.0015 (6)
C15	0.0192 (6)	0.0267 (7)	0.0317 (7)	−0.0038 (5)	0.0050 (5)	−0.0056 (6)
C16	0.0283 (7)	0.0292 (7)	0.0224 (6)	−0.0044 (6)	0.0028 (5)	0.0032 (6)
C17	0.0210 (6)	0.0216 (6)	0.0230 (6)	0.0005 (5)	−0.0024 (5)	0.0019 (5)
C18	0.0166 (6)	0.0252 (7)	0.0209 (6)	0.0059 (5)	0.0013 (5)	−0.0032 (5)
C19	0.0264 (7)	0.0326 (8)	0.0260 (7)	−0.0002 (6)	−0.0008 (6)	−0.0065 (6)
C20	0.0332 (8)	0.0514 (10)	0.0275 (8)	0.0014 (8)	−0.0042 (6)	−0.0121 (7)
C21	0.0321 (8)	0.0627 (12)	0.0202 (7)	0.0069 (8)	−0.0008 (6)	−0.0020 (7)
C22	0.0274 (7)	0.0458 (9)	0.0267 (7)	0.0042 (7)	0.0053 (6)	0.0054 (7)
C23	0.0204 (6)	0.0311 (7)	0.0250 (7)	0.0025 (6)	0.0020 (5)	−0.0006 (6)

### (1R,2S,4r)-1,2,4-Triphenylcyclopentane-1,2-diol (I) Geometric parameters (Å, º)

**Table:**

O1—C1	1.4426 (14)	C10—C11	1.387 (2)
O1—H1	0.87 (2)	C10—H10	0.97 (2)
O2—C2	1.4343 (15)	C11—H11	0.98 (2)
O2—H2	0.86 (2)	C12—C17	1.3898 (18)
C1—C6	1.5162 (17)	C12—C13	1.3985 (17)
C1—C5	1.5274 (18)	C13—C14	1.3895 (19)
C1—C2	1.6058 (17)	C13—H13	0.978 (19)
C2—C12	1.5230 (17)	C14—C15	1.387 (2)
C2—C3	1.5531 (18)	C14—H14	0.995 (19)
C3—C4	1.5319 (18)	C15—C16	1.379 (2)
C3—H3A	1.005 (17)	C15—H15	0.972 (19)
C3—H3B	0.990 (17)	C16—C17	1.395 (2)
C4—C18	1.5142 (18)	C16—H16	0.980 (19)
C4—C5	1.5369 (17)	C17—H17	0.94 (2)
C4—H4	1.002 (16)	C18—C19	1.389 (2)
C5—H5A	0.959 (17)	C18—C23	1.398 (2)
C5—H5B	0.990 (16)	C19—C20	1.399 (2)
C6—C7	1.3883 (18)	C19—H19	0.950 (19)
C6—C11	1.3930 (19)	C20—C21	1.382 (3)
C7—C8	1.391 (2)	C20—H20	0.96 (2)
C7—H7	0.973 (19)	C21—C22	1.383 (3)
C8—C9	1.382 (3)	C21—H21	0.98 (2)
C8—H8	0.98 (2)	C22—C23	1.386 (2)
C9—C10	1.384 (3)	C22—H22	0.97 (2)
C9—H9	0.98 (2)	C23—H23	0.989 (18)
			
C1—O1—H1	108.6 (13)	C10—C9—H9	120.1 (13)
C2—O2—H2	104.6 (14)	C9—C10—C11	120.21 (16)
O1—C1—C6	108.72 (10)	C9—C10—H10	120.1 (13)
O1—C1—C5	107.93 (10)	C11—C10—H10	119.6 (13)
C6—C1—C5	116.62 (10)	C10—C11—C6	120.70 (15)
O1—C1—C2	103.73 (9)	C10—C11—H11	119.3 (11)
C6—C1—C2	115.50 (10)	C6—C11—H11	120.0 (11)
C5—C1—C2	103.36 (9)	C17—C12—C13	118.02 (12)
O2—C2—C12	108.25 (10)	C17—C12—C2	120.98 (11)
O2—C2—C3	110.39 (10)	C13—C12—C2	120.97 (11)
C12—C2—C3	110.82 (10)	C14—C13—C12	120.68 (13)
O2—C2—C1	108.74 (9)	C14—C13—H13	119.1 (11)
C12—C2—C1	114.61 (10)	C12—C13—H13	120.2 (11)
C3—C2—C1	103.96 (10)	C15—C14—C13	120.60 (13)
C4—C3—C2	106.10 (10)	C15—C14—H14	119.7 (11)
C4—C3—H3A	109.7 (10)	C13—C14—H14	119.7 (11)
C2—C3—H3A	109.0 (10)	C16—C15—C14	119.25 (13)
C4—C3—H3B	113.8 (10)	C16—C15—H15	120.4 (11)
C2—C3—H3B	110.3 (10)	C14—C15—H15	120.3 (11)
H3A—C3—H3B	107.9 (14)	C15—C16—C17	120.29 (13)
C18—C4—C3	117.40 (11)	C15—C16—H16	120.7 (11)
C18—C4—C5	114.16 (11)	C17—C16—H16	119.0 (11)
C3—C4—C5	100.58 (10)	C12—C17—C16	121.12 (13)
C18—C4—H4	107.3 (9)	C12—C17—H17	119.1 (12)
C3—C4—H4	108.8 (9)	C16—C17—H17	119.7 (12)
C5—C4—H4	108.2 (9)	C19—C18—C23	118.32 (13)
C1—C5—C4	103.31 (10)	C19—C18—C4	123.22 (13)
C1—C5—H5A	111.9 (10)	C23—C18—C4	118.45 (12)
C4—C5—H5A	113.2 (10)	C18—C19—C20	120.45 (15)
C1—C5—H5B	113.1 (9)	C18—C19—H19	120.0 (11)
C4—C5—H5B	109.4 (9)	C20—C19—H19	119.5 (11)
H5A—C5—H5B	106.1 (14)	C21—C20—C19	120.40 (16)
C7—C6—C11	118.68 (12)	C21—C20—H20	121.6 (13)
C7—C6—C1	122.04 (12)	C19—C20—H20	118.0 (13)
C11—C6—C1	119.24 (12)	C20—C21—C22	119.62 (15)
C6—C7—C8	120.50 (14)	C20—C21—H21	119.1 (12)
C6—C7—H7	119.1 (11)	C22—C21—H21	121.3 (12)
C8—C7—H7	120.4 (11)	C21—C22—C23	120.09 (16)
C9—C8—C7	120.38 (15)	C21—C22—H22	119.7 (12)
C9—C8—H8	120.9 (12)	C23—C22—H22	120.2 (12)
C7—C8—H8	118.7 (12)	C22—C23—C18	121.12 (14)
C8—C9—C10	119.54 (14)	C22—C23—H23	118.8 (10)
C8—C9—H9	120.4 (13)	C18—C23—H23	120.1 (10)
			
O1—C1—C2—O2	−18.78 (12)	C9—C10—C11—C6	0.2 (3)
C6—C1—C2—O2	100.08 (12)	C7—C6—C11—C10	−0.3 (2)
C5—C1—C2—O2	−131.34 (10)	C1—C6—C11—C10	−178.18 (14)
O1—C1—C2—C12	−140.05 (10)	O2—C2—C12—C17	8.66 (16)
C6—C1—C2—C12	−21.20 (15)	C3—C2—C12—C17	−112.53 (13)
C5—C1—C2—C12	107.38 (11)	C1—C2—C12—C17	130.20 (12)
O1—C1—C2—C3	98.83 (11)	O2—C2—C12—C13	−173.27 (11)
C6—C1—C2—C3	−142.31 (11)	C3—C2—C12—C13	65.54 (15)
C5—C1—C2—C3	−13.74 (12)	C1—C2—C12—C13	−51.72 (16)
O2—C2—C3—C4	100.69 (12)	C17—C12—C13—C14	−1.9 (2)
C12—C2—C3—C4	−139.38 (10)	C2—C12—C13—C14	179.92 (12)
C1—C2—C3—C4	−15.77 (12)	C12—C13—C14—C15	1.6 (2)
C2—C3—C4—C18	163.45 (10)	C13—C14—C15—C16	0.1 (2)
C2—C3—C4—C5	39.00 (12)	C14—C15—C16—C17	−1.5 (2)
O1—C1—C5—C4	−71.24 (12)	C13—C12—C17—C16	0.62 (19)
C6—C1—C5—C4	166.10 (10)	C2—C12—C17—C16	178.74 (12)
C2—C1—C5—C4	38.23 (11)	C15—C16—C17—C12	1.1 (2)
C18—C4—C5—C1	−174.72 (10)	C3—C4—C18—C19	7.85 (19)
C3—C4—C5—C1	−48.08 (12)	C5—C4—C18—C19	125.18 (14)
O1—C1—C6—C7	−121.11 (13)	C3—C4—C18—C23	−173.66 (12)
C5—C1—C6—C7	1.13 (17)	C5—C4—C18—C23	−56.33 (16)
C2—C1—C6—C7	122.83 (13)	C23—C18—C19—C20	−0.3 (2)
O1—C1—C6—C11	56.65 (15)	C4—C18—C19—C20	178.23 (14)
C5—C1—C6—C11	178.89 (12)	C18—C19—C20—C21	−0.3 (3)
C2—C1—C6—C11	−59.41 (16)	C19—C20—C21—C22	1.1 (3)
C11—C6—C7—C8	0.3 (2)	C20—C21—C22—C23	−1.4 (3)
C1—C6—C7—C8	178.06 (13)	C21—C22—C23—C18	0.8 (2)
C6—C7—C8—C9	−0.1 (2)	C19—C18—C23—C22	0.0 (2)
C7—C8—C9—C10	0.0 (3)	C4—C18—C23—C22	−178.55 (13)
C8—C9—C10—C11	−0.1 (3)		

### (1R,2S,4r)-1,2,4-Triphenylcyclopentane-1,2-diol (I) Hydrogen-bond geometry (Å, º)

**Table:**

*D*—H···*A*	*D*—H	H···*A*	*D*···*A*	*D*—H···*A*
O1—H1···O2^i^	0.87 (2)	1.89 (2)	2.7509 (13)	173.2 (19)
O2—H2···O1	0.86 (2)	1.80 (2)	2.4510 (14)	131.0 (19)

Symmetry code: (i) −*x*+1/2, *y*−1/2, *z*.

### (1R,2S,4r)-4-(2-Methoxyphenyl)-1,2-diphenylcyclopentane-1,2-diol (II) Crystal data

**Table:**

C_24_H_24_O_3_	*Z* = 6
*M**_r_* = 360.43	*F*(000) = 1152
Triclinic, *P*1	*D*_x_ = 1.252 Mg m^−^^3^
*a* = 11.4136 (6) Å	Mo *K*α radiation, λ = 0.71073 Å
*b* = 14.0145 (7) Å	Cell parameters from 8113 reflections
*c* = 19.0339 (10) Å	θ = 2.4–29.8°
α = 92.3394 (18)°	µ = 0.08 mm^−^^1^
β = 101.5461 (17)°	*T* = 150 K
γ = 105.0129 (19)°	Prism, colourless
*V* = 2867.3 (3) Å^3^	0.50 × 0.20 × 0.10 mm

### (1R,2S,4r)-4-(2-Methoxyphenyl)-1,2-diphenylcyclopentane-1,2-diol (II) Data collection

**Table:**

Bruker SMART APEXII diffractometer	11202 independent reflections
Radiation source: fine-focus sealed tube	8043 reflections with *I* > 2σ(*I*)
Graphite monochromator	*R*_int_ = 0.039
ω scans	θ_max_ = 26.0°, θ_min_ = 2.0°
Absorption correction: multi-scan (SADABS; Krause *et al.*, 2015)	*h* = −14→14
*T*_min_ = 0.856, *T*_max_ = 0.928	*k* = −16→17
31335 measured reflections	*l* = −23→22

### (1R,2S,4r)-4-(2-Methoxyphenyl)-1,2-diphenylcyclopentane-1,2-diol (II) Refinement

**Table:**

Refinement on *F*^2^	Secondary atom site location: difference Fourier map
Least-squares matrix: full	Hydrogen site location: mixed
*R*[*F*^2^ > 2σ(*F*^2^)] = 0.051	H atoms treated by a mixture of independent and constrained refinement
*wR*(*F*^2^) = 0.136	*w* = 1/[σ^2^(*F*_o_^2^) + (0.062*P*)^2^ + 0.7914*P*] where *P* = (*F*_o_^2^ + 2*F*_c_^2^)/3
*S* = 1.04	(Δ/σ)_max_ < 0.001
11202 reflections	Δρ_max_ = 0.36 e Å^−^^3^
758 parameters	Δρ_min_ = −0.24 e Å^−^^3^
0 restraints	Extinction correction: SHELXL2017 (Sheldrick, 2015a), Fc^*^=kFc[1+0.001xFc^2^λ^3^/sin(2θ)]^-1/4^
Primary atom site location: structure-invariant direct methods	Extinction coefficient: 0.0066 (10)

### (1R,2S,4r)-4-(2-Methoxyphenyl)-1,2-diphenylcyclopentane-1,2-diol (II) Special details

**Table:**

Geometry. All esds (except the esd in the dihedral angle between two l.s. planes) are estimated using the full covariance matrix. The cell esds are taken into account individually in the estimation of esds in distances, angles and torsion angles; correlations between esds in cell parameters are only used when they are defined by crystal symmetry. An approximate (isotropic) treatment of cell esds is used for estimating esds involving l.s. planes.
Refinement. Refinement of F^2^ against ALL reflections. The weighted R-factor wR and goodness of fit S are based on F^2^, conventional R-factors R are based on F, with F set to zero for negative F^2^. The threshold expression of F^2^ > 2sigma(F^2^) is used only for calculating R-factors(gt) etc. and is not relevant to the choice of reflections for refinement. R-factors based on F^2^ are statistically about twice as large as those based on F, and R- factors based on ALL data will be even larger.

### (1R,2S,4r)-4-(2-Methoxyphenyl)-1,2-diphenylcyclopentane-1,2-diol (II) Fractional atomic coordinates and isotropic or equivalent isotropic displacement parameters (Å^2^)

**Table:**

	*x*	*y*	*z*	*U*_iso_*/*U*_eq_	
O1A	0.14728 (13)	0.13460 (10)	0.67974 (7)	0.0287 (3)	
H1A	0.102 (3)	0.079 (2)	0.6584 (15)	0.072 (9)*	
O2A	0.35980 (13)	0.26985 (10)	0.71380 (7)	0.0295 (3)	
H2A	0.301 (2)	0.2451 (19)	0.6748 (14)	0.056 (8)*	
C1A	0.22327 (18)	0.12411 (13)	0.74694 (10)	0.0256 (4)	
C2A	0.31467 (17)	0.22962 (13)	0.77471 (10)	0.0238 (4)	
C3A	0.42356 (18)	0.20437 (14)	0.82332 (10)	0.0270 (4)	
H3AA	0.402647	0.184037	0.869502	0.032*	
H3AB	0.498165	0.262023	0.833524	0.032*	
C4A	0.44566 (19)	0.11847 (15)	0.78056 (11)	0.0326 (5)	
H4A	0.495937	0.147876	0.745420	0.039*	
C5A	0.31299 (19)	0.06137 (15)	0.73604 (12)	0.0355 (5)	
H5AA	0.314106	0.052394	0.684338	0.043*	
H5AB	0.285661	−0.004918	0.753057	0.043*	
C6A	0.14252 (18)	0.08553 (13)	0.79971 (10)	0.0278 (4)	
C7A	0.1801 (2)	0.03115 (15)	0.85577 (12)	0.0417 (6)	
H7A	0.258336	0.017061	0.861285	0.050*	
C8A	0.1053 (3)	−0.00218 (19)	0.90302 (14)	0.0575 (7)	
H8A	0.132981	−0.038195	0.941167	0.069*	
C9A	−0.0091 (3)	0.01592 (19)	0.89579 (14)	0.0567 (7)	
H9A	−0.060895	−0.008400	0.928134	0.068*	
C10A	−0.0481 (2)	0.06996 (16)	0.84087 (12)	0.0433 (6)	
H10A	−0.127221	0.082528	0.835362	0.052*	
C11A	0.02736 (19)	0.10568 (14)	0.79406 (11)	0.0306 (5)	
H11A	0.000747	0.144447	0.757541	0.037*	
C12A	0.26047 (16)	0.30269 (12)	0.80976 (10)	0.0222 (4)	
C13A	0.20814 (18)	0.36708 (14)	0.76830 (11)	0.0292 (4)	
H13A	0.203599	0.362933	0.717838	0.035*	
C14A	0.1626 (2)	0.43709 (15)	0.79939 (12)	0.0358 (5)	
H14A	0.126196	0.479605	0.770007	0.043*	
C15A	0.1697 (2)	0.44537 (15)	0.87243 (12)	0.0374 (5)	
H15A	0.140423	0.494505	0.893829	0.045*	
C16A	0.2199 (2)	0.38141 (16)	0.91441 (11)	0.0369 (5)	
H16A	0.224158	0.386125	0.964841	0.044*	
C17A	0.26397 (19)	0.31062 (14)	0.88348 (10)	0.0297 (4)	
H17A	0.297182	0.266636	0.912960	0.036*	
C18A	0.51525 (19)	0.05483 (14)	0.82424 (12)	0.0343 (5)	
C19A	0.5404 (2)	0.06080 (17)	0.89954 (12)	0.0438 (6)	
H19A	0.511702	0.106584	0.925091	0.053*	
C20A	0.6054 (2)	0.00245 (18)	0.93782 (15)	0.0520 (6)	
H20A	0.619549	0.007599	0.988917	0.062*	
C21A	0.6495 (2)	−0.06274 (17)	0.90258 (15)	0.0506 (7)	
H21A	0.694726	−0.102728	0.929014	0.061*	
C22A	0.6279 (2)	−0.07035 (16)	0.82751 (15)	0.0451 (6)	
H22A	0.659841	−0.114413	0.802520	0.054*	
C23A	0.5596 (2)	−0.01324 (15)	0.78987 (13)	0.0390 (5)	
O3A	0.53096 (16)	−0.01728 (11)	0.71594 (9)	0.0487 (4)	
C24A	0.5601 (3)	−0.09494 (19)	0.67718 (15)	0.0593 (7)	
H24A	0.531866	−0.092688	0.625301	0.089*	
H24B	0.518130	−0.159399	0.691013	0.089*	
H24C	0.650153	−0.085771	0.688765	0.089*	
O1B	0.72932 (14)	0.59540 (10)	0.70807 (7)	0.0325 (3)	
H1B	0.667 (3)	0.551 (2)	0.6768 (16)	0.075 (9)*	
O2B	0.54682 (14)	0.44673 (10)	0.71982 (7)	0.0300 (3)	
H2B	0.496 (2)	0.3930 (18)	0.7227 (12)	0.040 (7)*	
C1B	0.68651 (18)	0.59658 (13)	0.77392 (10)	0.0248 (4)	
C2B	0.62450 (17)	0.48732 (13)	0.78901 (9)	0.0239 (4)	
C3B	0.54589 (17)	0.50509 (14)	0.84122 (10)	0.0253 (4)	
H3BA	0.598426	0.529602	0.889875	0.030*	
H3BB	0.482352	0.443150	0.844548	0.030*	
C4B	0.48449 (17)	0.58350 (13)	0.80977 (10)	0.0264 (4)	
H4B	0.407801	0.547362	0.773456	0.032*	
C5B	0.57701 (18)	0.64378 (14)	0.76731 (10)	0.0282 (4)	
H5BA	0.535520	0.641122	0.716063	0.034*	
H5BB	0.607415	0.714096	0.787718	0.034*	
C6B	0.79924 (18)	0.64960 (13)	0.83207 (10)	0.0279 (4)	
C7B	0.7894 (2)	0.70761 (14)	0.89084 (11)	0.0330 (5)	
H7B	0.710772	0.716166	0.894287	0.040*	
C8B	0.8941 (2)	0.75301 (16)	0.94442 (12)	0.0463 (6)	
H8B	0.886614	0.792497	0.984190	0.056*	
C9B	1.0082 (2)	0.74091 (17)	0.94001 (15)	0.0530 (7)	
H9B	1.079536	0.772039	0.976589	0.064*	
C10B	1.0188 (2)	0.68325 (18)	0.88210 (15)	0.0515 (7)	
H10B	1.097535	0.674489	0.879250	0.062*	
C11B	0.91550 (19)	0.63821 (16)	0.82828 (13)	0.0389 (5)	
H11B	0.923985	0.599297	0.788524	0.047*	
C12B	0.71105 (18)	0.42414 (13)	0.81557 (10)	0.0255 (4)	
C13B	0.77772 (19)	0.43761 (15)	0.88672 (11)	0.0319 (5)	
H13B	0.770028	0.488052	0.918968	0.038*	
C14B	0.8550 (2)	0.37885 (16)	0.91130 (13)	0.0419 (5)	
H14B	0.900588	0.389782	0.959841	0.050*	
C15B	0.8658 (2)	0.30442 (17)	0.86533 (13)	0.0450 (6)	
H15B	0.918043	0.263540	0.882187	0.054*	
C16B	0.8005 (2)	0.28981 (17)	0.79511 (13)	0.0436 (6)	
H16B	0.807719	0.238573	0.763358	0.052*	
C17B	0.7242 (2)	0.34933 (15)	0.77018 (11)	0.0338 (5)	
H17B	0.680232	0.338749	0.721309	0.041*	
C18B	0.44390 (17)	0.64346 (15)	0.86390 (11)	0.0317 (5)	
C19B	0.4625 (2)	0.63124 (17)	0.93707 (12)	0.0405 (5)	
H19B	0.506993	0.585623	0.955254	0.049*	
C20B	0.4182 (2)	0.6835 (2)	0.98427 (14)	0.0542 (7)	
H20B	0.430586	0.672705	1.033846	0.065*	
C21B	0.3564 (2)	0.7507 (2)	0.95862 (15)	0.0593 (8)	
H21B	0.326291	0.787077	0.990813	0.071*	
C22B	0.3371 (2)	0.76666 (18)	0.88654 (16)	0.0520 (7)	
H22B	0.295509	0.814533	0.869395	0.062*	
C23B	0.37916 (19)	0.71186 (15)	0.83939 (12)	0.0375 (5)	
O3B	0.35836 (14)	0.71818 (10)	0.76626 (9)	0.0427 (4)	
C24B	0.2835 (2)	0.78050 (18)	0.73784 (17)	0.0621 (8)	
H47D	0.278101	0.780666	0.685806	0.093*	
H47E	0.320979	0.848241	0.761150	0.093*	
H47F	0.200007	0.755498	0.746985	0.093*	
O1C	0.83623 (13)	0.78847 (10)	0.67276 (7)	0.0293 (3)	
H1C	0.810 (2)	0.7296 (19)	0.6878 (12)	0.046 (7)*	
O2C	1.00942 (13)	0.92850 (10)	0.64099 (8)	0.0287 (3)	
H2C	0.980 (2)	0.9079 (18)	0.6767 (14)	0.051 (8)*	
C1C	0.85364 (17)	0.77202 (13)	0.60124 (9)	0.0224 (4)	
C2C	0.91281 (17)	0.87784 (13)	0.57931 (9)	0.0237 (4)	
C3C	0.97825 (17)	0.85444 (13)	0.52145 (10)	0.0241 (4)	
H3CA	1.041397	0.914312	0.514056	0.029*	
H3CB	0.917915	0.829337	0.475174	0.029*	
C4C	1.04010 (17)	0.77404 (13)	0.55018 (10)	0.0235 (4)	
H4C	1.122322	0.809106	0.582118	0.028*	
C5C	0.95607 (17)	0.71870 (13)	0.59948 (10)	0.0246 (4)	
H5CA	0.918233	0.648414	0.579981	0.029*	
H5CB	1.006011	0.720663	0.648723	0.029*	
C6C	0.72964 (17)	0.71953 (12)	0.55061 (9)	0.0225 (4)	
C7C	0.72397 (18)	0.66404 (13)	0.48685 (9)	0.0246 (4)	
H7C	0.798411	0.656154	0.475394	0.029*	
C8C	0.61058 (19)	0.62020 (15)	0.43994 (11)	0.0320 (5)	
H8C	0.608086	0.582538	0.396759	0.038*	
C9C	0.5012 (2)	0.63106 (15)	0.45574 (12)	0.0372 (5)	
H9C	0.423755	0.601828	0.423356	0.045*	
C10C	0.50610 (19)	0.68497 (16)	0.51921 (12)	0.0376 (5)	
H10C	0.431508	0.692317	0.530778	0.045*	
C11C	0.61908 (18)	0.72833 (14)	0.56603 (11)	0.0301 (4)	
H11C	0.620852	0.764836	0.609582	0.036*	
C12C	0.82353 (17)	0.94100 (13)	0.55790 (10)	0.0249 (4)	
C13C	0.74574 (19)	0.92723 (15)	0.48961 (11)	0.0304 (4)	
H13C	0.746555	0.876044	0.455561	0.036*	
C14C	0.6674 (2)	0.98699 (16)	0.47066 (12)	0.0379 (5)	
H14C	0.615020	0.976387	0.423859	0.045*	
C15C	0.6646 (2)	1.06201 (16)	0.51930 (13)	0.0417 (6)	
H15C	0.611136	1.103224	0.506051	0.050*	
C16C	0.7400 (2)	1.07624 (16)	0.58697 (14)	0.0438 (6)	
H16C	0.738493	1.127377	0.620812	0.053*	
C17C	0.81854 (19)	1.01628 (15)	0.60615 (12)	0.0350 (5)	
H17C	0.869919	1.026903	0.653229	0.042*	
C18C	1.06489 (17)	0.70730 (13)	0.49336 (10)	0.0251 (4)	
C19C	1.03585 (18)	0.71550 (14)	0.41988 (10)	0.0285 (4)	
H19C	0.994730	0.763803	0.403130	0.034*	
C20C	1.06512 (19)	0.65517 (16)	0.37003 (11)	0.0343 (5)	
H20C	1.044696	0.662858	0.320080	0.041*	
C21C	1.12357 (19)	0.58454 (15)	0.39328 (11)	0.0348 (5)	
H21C	1.142862	0.542847	0.359249	0.042*	
C22C	1.15471 (18)	0.57361 (14)	0.46625 (11)	0.0313 (5)	
H22C	1.194781	0.524405	0.482294	0.038*	
C23C	1.12666 (18)	0.63544 (14)	0.51566 (10)	0.0271 (4)	
O3C	1.15809 (14)	0.63245 (10)	0.58874 (7)	0.0342 (3)	
C24C	1.2122 (2)	0.55618 (16)	0.61470 (12)	0.0391 (5)	
H24G	1.229764	0.562486	0.667487	0.059*	
H24H	1.289720	0.562436	0.598343	0.059*	
H24I	1.154227	0.491171	0.596126	0.059*	

### (1R,2S,4r)-4-(2-Methoxyphenyl)-1,2-diphenylcyclopentane-1,2-diol (II) Atomic displacement parameters (Å^2^)

**Table:**

	*U*^11^	*U*^22^	*U*^33^	*U*^12^	*U*^13^	*U*^23^
O1A	0.0318 (8)	0.0254 (7)	0.0242 (7)	0.0063 (6)	−0.0022 (6)	−0.0010 (6)
O2A	0.0326 (8)	0.0310 (7)	0.0230 (7)	0.0055 (6)	0.0067 (6)	0.0013 (6)
C1A	0.0277 (10)	0.0206 (9)	0.0257 (9)	0.0081 (8)	−0.0019 (8)	−0.0014 (8)
C2A	0.0257 (10)	0.0220 (9)	0.0220 (9)	0.0039 (8)	0.0047 (8)	0.0014 (7)
C3A	0.0255 (10)	0.0258 (10)	0.0287 (10)	0.0075 (8)	0.0038 (8)	−0.0003 (8)
C4A	0.0314 (11)	0.0308 (11)	0.0361 (11)	0.0103 (9)	0.0067 (9)	−0.0002 (9)
C5A	0.0357 (12)	0.0299 (11)	0.0388 (12)	0.0137 (9)	−0.0007 (9)	−0.0065 (9)
C6A	0.0324 (11)	0.0156 (9)	0.0294 (10)	0.0023 (8)	−0.0014 (8)	0.0017 (8)
C7A	0.0379 (13)	0.0329 (11)	0.0466 (13)	0.0018 (10)	−0.0010 (11)	0.0178 (10)
C8A	0.0536 (17)	0.0531 (15)	0.0540 (15)	−0.0015 (13)	0.0012 (13)	0.0286 (13)
C9A	0.0576 (17)	0.0534 (15)	0.0511 (15)	−0.0071 (13)	0.0204 (13)	0.0177 (13)
C10A	0.0410 (13)	0.0377 (12)	0.0474 (13)	0.0014 (10)	0.0132 (11)	0.0037 (11)
C11A	0.0356 (11)	0.0205 (9)	0.0323 (11)	0.0033 (8)	0.0048 (9)	0.0026 (8)
C12A	0.0193 (9)	0.0178 (8)	0.0265 (9)	0.0006 (7)	0.0041 (8)	0.0001 (7)
C13A	0.0306 (11)	0.0288 (10)	0.0276 (10)	0.0085 (9)	0.0042 (8)	0.0028 (8)
C14A	0.0361 (12)	0.0292 (11)	0.0445 (12)	0.0145 (9)	0.0060 (10)	0.0081 (9)
C15A	0.0372 (12)	0.0299 (11)	0.0491 (13)	0.0132 (9)	0.0145 (10)	−0.0028 (10)
C16A	0.0453 (13)	0.0397 (12)	0.0285 (11)	0.0114 (10)	0.0159 (10)	−0.0008 (9)
C17A	0.0343 (11)	0.0275 (10)	0.0291 (10)	0.0102 (9)	0.0086 (9)	0.0055 (8)
C18A	0.0247 (10)	0.0250 (10)	0.0527 (13)	0.0058 (8)	0.0084 (10)	0.0046 (9)
C19A	0.0422 (13)	0.0419 (13)	0.0437 (13)	0.0168 (11)	−0.0042 (11)	−0.0029 (11)
C20A	0.0526 (15)	0.0415 (13)	0.0604 (16)	0.0182 (12)	0.0019 (13)	0.0038 (12)
C21A	0.0363 (13)	0.0368 (13)	0.0756 (18)	0.0156 (11)	−0.0034 (12)	0.0113 (12)
C22A	0.0370 (13)	0.0309 (11)	0.0714 (17)	0.0156 (10)	0.0123 (12)	0.0049 (11)
C23A	0.0310 (12)	0.0296 (11)	0.0590 (15)	0.0071 (9)	0.0165 (11)	0.0087 (10)
O3A	0.0668 (11)	0.0407 (9)	0.0534 (10)	0.0282 (8)	0.0288 (9)	0.0082 (8)
C24A	0.085 (2)	0.0468 (15)	0.0678 (17)	0.0359 (14)	0.0431 (16)	0.0106 (13)
O1B	0.0421 (9)	0.0314 (8)	0.0265 (7)	0.0082 (7)	0.0142 (7)	0.0094 (6)
O2B	0.0366 (8)	0.0254 (7)	0.0219 (7)	0.0020 (7)	0.0007 (6)	0.0031 (6)
C1B	0.0299 (10)	0.0239 (9)	0.0218 (9)	0.0069 (8)	0.0079 (8)	0.0077 (8)
C2B	0.0253 (10)	0.0245 (9)	0.0190 (9)	0.0036 (8)	0.0020 (8)	0.0038 (7)
C3B	0.0228 (10)	0.0268 (9)	0.0238 (9)	0.0028 (8)	0.0040 (8)	0.0042 (8)
C4B	0.0222 (10)	0.0257 (10)	0.0276 (10)	0.0037 (8)	0.0008 (8)	0.0007 (8)
C5B	0.0290 (11)	0.0252 (10)	0.0283 (10)	0.0067 (8)	0.0012 (8)	0.0061 (8)
C6B	0.0264 (10)	0.0235 (9)	0.0315 (10)	0.0022 (8)	0.0055 (8)	0.0109 (8)
C7B	0.0352 (12)	0.0269 (10)	0.0316 (11)	0.0018 (9)	0.0034 (9)	0.0050 (9)
C8B	0.0531 (16)	0.0318 (12)	0.0383 (12)	−0.0044 (11)	−0.0050 (11)	0.0047 (10)
C9B	0.0378 (14)	0.0368 (13)	0.0629 (17)	−0.0092 (11)	−0.0157 (12)	0.0177 (12)
C10B	0.0265 (12)	0.0425 (13)	0.0800 (19)	0.0035 (10)	0.0038 (12)	0.0201 (14)
C11B	0.0295 (12)	0.0331 (11)	0.0535 (14)	0.0051 (9)	0.0101 (10)	0.0152 (10)
C12B	0.0272 (10)	0.0220 (9)	0.0281 (10)	0.0033 (8)	0.0108 (8)	0.0087 (8)
C13B	0.0339 (11)	0.0291 (10)	0.0329 (11)	0.0101 (9)	0.0051 (9)	0.0067 (9)
C14B	0.0397 (13)	0.0437 (13)	0.0422 (13)	0.0165 (11)	0.0010 (10)	0.0119 (11)
C15B	0.0442 (14)	0.0432 (13)	0.0575 (15)	0.0252 (11)	0.0143 (12)	0.0177 (11)
C16B	0.0499 (14)	0.0401 (13)	0.0519 (14)	0.0224 (11)	0.0234 (12)	0.0070 (11)
C17B	0.0385 (12)	0.0334 (11)	0.0334 (11)	0.0109 (9)	0.0145 (9)	0.0071 (9)
C18B	0.0171 (9)	0.0313 (10)	0.0404 (11)	0.0003 (8)	0.0024 (8)	−0.0065 (9)
C19B	0.0284 (11)	0.0497 (13)	0.0378 (12)	0.0044 (10)	0.0053 (9)	−0.0084 (10)
C20B	0.0309 (13)	0.0759 (18)	0.0469 (14)	0.0026 (13)	0.0090 (11)	−0.0200 (13)
C21B	0.0296 (13)	0.0715 (18)	0.0677 (18)	0.0051 (13)	0.0097 (12)	−0.0353 (15)
C22B	0.0239 (12)	0.0432 (13)	0.084 (2)	0.0084 (10)	0.0054 (12)	−0.0188 (13)
C23B	0.0214 (10)	0.0333 (11)	0.0500 (13)	0.0019 (9)	0.0000 (9)	−0.0080 (10)
O3B	0.0346 (8)	0.0324 (8)	0.0584 (10)	0.0164 (7)	−0.0051 (7)	0.0023 (7)
C24B	0.0457 (15)	0.0407 (13)	0.091 (2)	0.0219 (12)	−0.0171 (14)	−0.0009 (14)
O1C	0.0412 (8)	0.0262 (7)	0.0194 (6)	0.0047 (6)	0.0095 (6)	0.0031 (6)
O2C	0.0288 (8)	0.0240 (7)	0.0265 (7)	0.0002 (6)	0.0001 (6)	−0.0013 (6)
C1C	0.0286 (10)	0.0207 (9)	0.0166 (8)	0.0040 (8)	0.0055 (7)	0.0028 (7)
C2C	0.0254 (10)	0.0200 (9)	0.0219 (9)	0.0009 (7)	0.0034 (8)	0.0001 (7)
C3C	0.0254 (10)	0.0220 (9)	0.0253 (9)	0.0044 (8)	0.0087 (8)	0.0074 (8)
C4C	0.0237 (10)	0.0222 (9)	0.0238 (9)	0.0050 (8)	0.0044 (8)	0.0046 (7)
C5C	0.0275 (10)	0.0226 (9)	0.0219 (9)	0.0052 (8)	0.0028 (8)	0.0052 (7)
C6C	0.0267 (10)	0.0170 (8)	0.0238 (9)	0.0034 (7)	0.0079 (8)	0.0055 (7)
C7C	0.0280 (10)	0.0232 (9)	0.0233 (9)	0.0072 (8)	0.0068 (8)	0.0039 (8)
C8C	0.0363 (12)	0.0303 (10)	0.0258 (10)	0.0071 (9)	0.0016 (9)	−0.0019 (8)
C9C	0.0270 (11)	0.0364 (11)	0.0399 (12)	0.0019 (9)	−0.0020 (9)	−0.0014 (10)
C10C	0.0250 (11)	0.0389 (12)	0.0487 (13)	0.0054 (9)	0.0124 (10)	0.0034 (10)
C11C	0.0321 (11)	0.0276 (10)	0.0306 (10)	0.0052 (9)	0.0112 (9)	−0.0003 (8)
C12C	0.0243 (10)	0.0189 (9)	0.0300 (10)	0.0008 (7)	0.0089 (8)	0.0041 (8)
C13C	0.0346 (11)	0.0303 (10)	0.0298 (10)	0.0126 (9)	0.0100 (9)	0.0046 (8)
C14C	0.0363 (12)	0.0428 (12)	0.0394 (12)	0.0174 (10)	0.0090 (10)	0.0123 (10)
C15C	0.0364 (12)	0.0345 (12)	0.0629 (15)	0.0186 (10)	0.0178 (12)	0.0114 (11)
C16C	0.0375 (13)	0.0321 (11)	0.0625 (16)	0.0106 (10)	0.0140 (12)	−0.0091 (11)
C17C	0.0307 (11)	0.0310 (11)	0.0402 (12)	0.0035 (9)	0.0087 (9)	−0.0063 (9)
C18C	0.0217 (10)	0.0245 (9)	0.0277 (10)	0.0028 (8)	0.0062 (8)	0.0052 (8)
C19C	0.0265 (10)	0.0297 (10)	0.0297 (10)	0.0070 (8)	0.0074 (8)	0.0066 (8)
C20C	0.0331 (11)	0.0415 (12)	0.0275 (10)	0.0072 (10)	0.0090 (9)	0.0035 (9)
C21C	0.0340 (12)	0.0366 (11)	0.0339 (11)	0.0082 (9)	0.0115 (9)	−0.0051 (9)
C22C	0.0267 (10)	0.0274 (10)	0.0408 (12)	0.0090 (8)	0.0080 (9)	0.0039 (9)
C23C	0.0249 (10)	0.0263 (10)	0.0287 (10)	0.0046 (8)	0.0056 (8)	0.0042 (8)
O3C	0.0447 (9)	0.0330 (8)	0.0287 (7)	0.0206 (7)	0.0034 (6)	0.0059 (6)
C24C	0.0460 (13)	0.0355 (12)	0.0407 (12)	0.0205 (10)	0.0065 (10)	0.0121 (10)

### (1R,2S,4r)-4-(2-Methoxyphenyl)-1,2-diphenylcyclopentane-1,2-diol (II) Geometric parameters (Å, º)

**Table:**

O1A—C1A	1.431 (2)	C11B—H11B	0.9500
O1A—H1A	0.85 (3)	C12B—C17B	1.388 (3)
O2A—C2A	1.439 (2)	C12B—C13B	1.393 (3)
O2A—H2A	0.88 (3)	C13B—C14B	1.385 (3)
C1A—C6A	1.519 (3)	C13B—H13B	0.9500
C1A—C5A	1.550 (3)	C14B—C15B	1.380 (3)
C1A—C2A	1.572 (2)	C14B—H14B	0.9500
C2A—C12A	1.521 (2)	C15B—C16B	1.372 (3)
C2A—C3A	1.523 (3)	C15B—H15B	0.9500
C3A—C4A	1.528 (3)	C16B—C17B	1.387 (3)
C3A—H3AA	0.9900	C16B—H16B	0.9500
C3A—H3AB	0.9900	C17B—H17B	0.9500
C4A—C18A	1.510 (3)	C18B—C19B	1.391 (3)
C4A—C5A	1.567 (3)	C18B—C23B	1.396 (3)
C4A—H4A	1.0000	C19B—C20B	1.385 (3)
C5A—H5AA	0.9900	C19B—H19B	0.9500
C5A—H5AB	0.9900	C20B—C21B	1.367 (4)
C6A—C7A	1.399 (3)	C20B—H20B	0.9500
C6A—C11A	1.399 (3)	C21B—C22B	1.382 (4)
C7A—C8A	1.376 (4)	C21B—H21B	0.9500
C7A—H7A	0.9500	C22B—C23B	1.390 (3)
C8A—C9A	1.375 (4)	C22B—H22B	0.9500
C8A—H8A	0.9500	C23B—O3B	1.375 (3)
C9A—C10A	1.385 (3)	O3B—C24B	1.423 (3)
C9A—H9A	0.9500	C24B—H47D	0.9800
C10A—C11A	1.381 (3)	C24B—H47E	0.9800
C10A—H10A	0.9500	C24B—H47F	0.9800
C11A—H11A	0.9500	O1C—C1C	1.433 (2)
C12A—C13A	1.394 (3)	O1C—H1C	0.88 (3)
C12A—C17A	1.395 (3)	O2C—C2C	1.451 (2)
C13A—C14A	1.387 (3)	O2C—H2C	0.85 (3)
C13A—H13A	0.9500	C1C—C6C	1.526 (2)
C14A—C15A	1.375 (3)	C1C—C5C	1.547 (3)
C14A—H14A	0.9500	C1C—C2C	1.573 (2)
C15A—C16A	1.383 (3)	C2C—C3C	1.518 (3)
C15A—H15A	0.9500	C2C—C12C	1.523 (3)
C16A—C17A	1.383 (3)	C3C—C4C	1.541 (2)
C16A—H16A	0.9500	C3C—H3CA	0.9900
C17A—H17A	0.9500	C3C—H3CB	0.9900
C18A—C23A	1.388 (3)	C4C—C18C	1.517 (3)
C18A—C19A	1.399 (3)	C4C—C5C	1.561 (3)
C19A—C20A	1.377 (3)	C4C—H4C	1.0000
C19A—H19A	0.9500	C5C—H5CA	0.9900
C20A—C21A	1.365 (3)	C5C—H5CB	0.9900
C20A—H20A	0.9500	C6C—C11C	1.386 (3)
C21A—C22A	1.395 (4)	C6C—C7C	1.395 (3)
C21A—H21A	0.9500	C7C—C8C	1.389 (3)
C22A—C23A	1.381 (3)	C7C—H7C	0.9500
C22A—H22A	0.9500	C8C—C9C	1.385 (3)
C23A—O3A	1.375 (3)	C8C—H8C	0.9500
O3A—C24A	1.435 (3)	C9C—C10C	1.382 (3)
C24A—H24A	0.9800	C9C—H9C	0.9500
C24A—H24B	0.9800	C10C—C11C	1.384 (3)
C24A—H24C	0.9800	C10C—H10C	0.9500
O1B—C1B	1.434 (2)	C11C—H11C	0.9500
O1B—H1B	0.90 (3)	C12C—C17C	1.390 (3)
O2B—C2B	1.433 (2)	C12C—C13C	1.394 (3)
O2B—H2B	0.83 (2)	C13C—C14C	1.384 (3)
C1B—C6B	1.517 (3)	C13C—H13C	0.9500
C1B—C5B	1.545 (3)	C14C—C15C	1.383 (3)
C1B—C2B	1.576 (3)	C14C—H14C	0.9500
C2B—C12B	1.518 (3)	C15C—C16C	1.373 (3)
C2B—C3B	1.519 (3)	C15C—H15C	0.9500
C3B—C4B	1.530 (3)	C16C—C17C	1.388 (3)
C3B—H3BA	0.9900	C16C—H16C	0.9500
C3B—H3BB	0.9900	C17C—H17C	0.9500
C4B—C18B	1.518 (3)	C18C—C19C	1.388 (3)
C4B—C5B	1.555 (3)	C18C—C23C	1.405 (3)
C4B—H4B	1.0000	C19C—C20C	1.391 (3)
C5B—H5BA	0.9900	C19C—H19C	0.9500
C5B—H5BB	0.9900	C20C—C21C	1.371 (3)
C6B—C11B	1.392 (3)	C20C—H20C	0.9500
C6B—C7B	1.396 (3)	C21C—C22C	1.388 (3)
C7B—C8B	1.392 (3)	C21C—H21C	0.9500
C7B—H7B	0.9500	C22C—C23C	1.391 (3)
C8B—C9B	1.374 (4)	C22C—H22C	0.9500
C8B—H8B	0.9500	C23C—O3C	1.370 (2)
C9B—C10B	1.383 (4)	O3C—C24C	1.425 (2)
C9B—H9B	0.9500	C24C—H24G	0.9800
C10B—C11B	1.384 (3)	C24C—H24H	0.9800
C10B—H10B	0.9500	C24C—H24I	0.9800
			
C1A—O1A—H1A	112.0 (19)	C10B—C11B—C6B	120.4 (2)
C2A—O2A—H2A	107.8 (17)	C10B—C11B—H11B	119.8
O1A—C1A—C6A	110.11 (15)	C6B—C11B—H11B	119.8
O1A—C1A—C5A	111.34 (15)	C17B—C12B—C13B	117.69 (18)
C6A—C1A—C5A	113.96 (16)	C17B—C12B—C2B	121.09 (17)
O1A—C1A—C2A	106.48 (14)	C13B—C12B—C2B	121.20 (17)
C6A—C1A—C2A	111.94 (15)	C14B—C13B—C12B	121.2 (2)
C5A—C1A—C2A	102.56 (15)	C14B—C13B—H13B	119.4
O2A—C2A—C12A	109.77 (14)	C12B—C13B—H13B	119.4
O2A—C2A—C3A	106.20 (15)	C15B—C14B—C13B	120.0 (2)
C12A—C2A—C3A	115.04 (15)	C15B—C14B—H14B	120.0
O2A—C2A—C1A	106.81 (14)	C13B—C14B—H14B	120.0
C12A—C2A—C1A	115.93 (15)	C16B—C15B—C14B	119.6 (2)
C3A—C2A—C1A	102.28 (14)	C16B—C15B—H15B	120.2
C2A—C3A—C4A	104.60 (15)	C14B—C15B—H15B	120.2
C2A—C3A—H3AA	110.8	C15B—C16B—C17B	120.5 (2)
C4A—C3A—H3AA	110.8	C15B—C16B—H16B	119.8
C2A—C3A—H3AB	110.8	C17B—C16B—H16B	119.8
C4A—C3A—H3AB	110.8	C16B—C17B—C12B	121.0 (2)
H3AA—C3A—H3AB	108.9	C16B—C17B—H17B	119.5
C18A—C4A—C3A	115.73 (17)	C12B—C17B—H17B	119.5
C18A—C4A—C5A	114.89 (17)	C19B—C18B—C23B	117.2 (2)
C3A—C4A—C5A	104.00 (16)	C19B—C18B—C4B	123.68 (18)
C18A—C4A—H4A	107.2	C23B—C18B—C4B	119.02 (19)
C3A—C4A—H4A	107.2	C20B—C19B—C18B	122.1 (2)
C5A—C4A—H4A	107.2	C20B—C19B—H19B	119.0
C1A—C5A—C4A	107.50 (15)	C18B—C19B—H19B	119.0
C1A—C5A—H5AA	110.2	C21B—C20B—C19B	119.2 (3)
C4A—C5A—H5AA	110.2	C21B—C20B—H20B	120.4
C1A—C5A—H5AB	110.2	C19B—C20B—H20B	120.4
C4A—C5A—H5AB	110.2	C20B—C21B—C22B	121.0 (2)
H5AA—C5A—H5AB	108.5	C20B—C21B—H21B	119.5
C7A—C6A—C11A	117.7 (2)	C22B—C21B—H21B	119.5
C7A—C6A—C1A	122.19 (19)	C21B—C22B—C23B	119.3 (2)
C11A—C6A—C1A	120.06 (16)	C21B—C22B—H22B	120.4
C8A—C7A—C6A	120.8 (2)	C23B—C22B—H22B	120.4
C8A—C7A—H7A	119.6	O3B—C23B—C22B	123.7 (2)
C6A—C7A—H7A	119.6	O3B—C23B—C18B	115.09 (18)
C9A—C8A—C7A	120.9 (2)	C22B—C23B—C18B	121.2 (2)
C9A—C8A—H8A	119.6	C23B—O3B—C24B	117.21 (19)
C7A—C8A—H8A	119.6	O3B—C24B—H47D	109.5
C8A—C9A—C10A	119.3 (2)	O3B—C24B—H47E	109.5
C8A—C9A—H9A	120.3	H47D—C24B—H47E	109.5
C10A—C9A—H9A	120.3	O3B—C24B—H47F	109.5
C11A—C10A—C9A	120.3 (2)	H47D—C24B—H47F	109.5
C11A—C10A—H10A	119.8	H47E—C24B—H47F	109.5
C9A—C10A—H10A	119.8	C1C—O1C—H1C	106.9 (15)
C10A—C11A—C6A	120.88 (19)	C2C—O2C—H2C	103.6 (18)
C10A—C11A—H11A	119.6	O1C—C1C—C6C	110.23 (15)
C6A—C11A—H11A	119.6	O1C—C1C—C5C	112.18 (14)
C13A—C12A—C17A	117.48 (17)	C6C—C1C—C5C	113.42 (14)
C13A—C12A—C2A	120.01 (16)	O1C—C1C—C2C	105.54 (14)
C17A—C12A—C2A	122.47 (16)	C6C—C1C—C2C	112.39 (14)
C14A—C13A—C12A	121.18 (18)	C5C—C1C—C2C	102.62 (14)
C14A—C13A—H13A	119.4	O2C—C2C—C3C	106.63 (15)
C12A—C13A—H13A	119.4	O2C—C2C—C12C	109.57 (14)
C15A—C14A—C13A	120.44 (19)	C3C—C2C—C12C	115.35 (15)
C15A—C14A—H14A	119.8	O2C—C2C—C1C	106.13 (14)
C13A—C14A—H14A	119.8	C3C—C2C—C1C	102.59 (14)
C14A—C15A—C16A	119.29 (19)	C12C—C2C—C1C	115.77 (15)
C14A—C15A—H15A	120.4	C2C—C3C—C4C	104.93 (14)
C16A—C15A—H15A	120.4	C2C—C3C—H3CA	110.8
C15A—C16A—C17A	120.44 (19)	C4C—C3C—H3CA	110.8
C15A—C16A—H16A	119.8	C2C—C3C—H3CB	110.8
C17A—C16A—H16A	119.8	C4C—C3C—H3CB	110.8
C16A—C17A—C12A	121.13 (18)	H3CA—C3C—H3CB	108.8
C16A—C17A—H17A	119.4	C18C—C4C—C3C	115.56 (15)
C12A—C17A—H17A	119.4	C18C—C4C—C5C	114.86 (15)
C23A—C18A—C19A	116.3 (2)	C3C—C4C—C5C	104.57 (14)
C23A—C18A—C4A	120.1 (2)	C18C—C4C—H4C	107.1
C19A—C18A—C4A	123.56 (19)	C3C—C4C—H4C	107.1
C20A—C19A—C18A	122.1 (2)	C5C—C4C—H4C	107.1
C20A—C19A—H19A	118.9	C1C—C5C—C4C	107.41 (14)
C18A—C19A—H19A	118.9	C1C—C5C—H5CA	110.2
C21A—C20A—C19A	120.2 (3)	C4C—C5C—H5CA	110.2
C21A—C20A—H20A	119.9	C1C—C5C—H5CB	110.2
C19A—C20A—H20A	119.9	C4C—C5C—H5CB	110.2
C20A—C21A—C22A	119.7 (2)	H5CA—C5C—H5CB	108.5
C20A—C21A—H21A	120.2	C11C—C6C—C7C	118.08 (17)
C22A—C21A—H21A	120.2	C11C—C6C—C1C	120.41 (16)
C23A—C22A—C21A	119.4 (2)	C7C—C6C—C1C	121.49 (17)
C23A—C22A—H22A	120.3	C8C—C7C—C6C	120.68 (18)
C21A—C22A—H22A	120.3	C8C—C7C—H7C	119.7
O3A—C23A—C22A	123.7 (2)	C6C—C7C—H7C	119.7
O3A—C23A—C18A	114.08 (19)	C9C—C8C—C7C	120.42 (19)
C22A—C23A—C18A	122.3 (2)	C9C—C8C—H8C	119.8
C23A—O3A—C24A	116.64 (18)	C7C—C8C—H8C	119.8
O3A—C24A—H24A	109.5	C10C—C9C—C8C	119.14 (19)
O3A—C24A—H24B	109.5	C10C—C9C—H9C	120.4
H24A—C24A—H24B	109.5	C8C—C9C—H9C	120.4
O3A—C24A—H24C	109.5	C9C—C10C—C11C	120.4 (2)
H24A—C24A—H24C	109.5	C9C—C10C—H10C	119.8
H24B—C24A—H24C	109.5	C11C—C10C—H10C	119.8
C1B—O1B—H1B	104.6 (19)	C10C—C11C—C6C	121.27 (19)
C2B—O2B—H2B	110.9 (16)	C10C—C11C—H11C	119.4
O1B—C1B—C6B	106.18 (15)	C6C—C11C—H11C	119.4
O1B—C1B—C5B	111.73 (14)	C17C—C12C—C13C	117.52 (18)
C6B—C1B—C5B	113.99 (15)	C17C—C12C—C2C	120.56 (17)
O1B—C1B—C2B	110.01 (14)	C13C—C12C—C2C	121.92 (16)
C6B—C1B—C2B	113.17 (14)	C14C—C13C—C12C	121.01 (19)
C5B—C1B—C2B	101.86 (15)	C14C—C13C—H13C	119.5
O2B—C2B—C12B	111.05 (15)	C12C—C13C—H13C	119.5
O2B—C2B—C3B	110.25 (15)	C15C—C14C—C13C	120.5 (2)
C12B—C2B—C3B	114.29 (15)	C15C—C14C—H14C	119.7
O2B—C2B—C1B	101.57 (14)	C13C—C14C—H14C	119.7
C12B—C2B—C1B	117.05 (15)	C16C—C15C—C14C	119.24 (19)
C3B—C2B—C1B	101.58 (14)	C16C—C15C—H15C	120.4
C2B—C3B—C4B	105.00 (15)	C14C—C15C—H15C	120.4
C2B—C3B—H3BA	110.7	C15C—C16C—C17C	120.3 (2)
C4B—C3B—H3BA	110.7	C15C—C16C—H16C	119.8
C2B—C3B—H3BB	110.7	C17C—C16C—H16C	119.8
C4B—C3B—H3BB	110.7	C16C—C17C—C12C	121.4 (2)
H3BA—C3B—H3BB	108.8	C16C—C17C—H17C	119.3
C18B—C4B—C3B	114.93 (16)	C12C—C17C—H17C	119.3
C18B—C4B—C5B	116.17 (16)	C19C—C18C—C23C	116.97 (17)
C3B—C4B—C5B	104.48 (15)	C19C—C18C—C4C	124.14 (16)
C18B—C4B—H4B	106.9	C23C—C18C—C4C	118.81 (16)
C3B—C4B—H4B	106.9	C18C—C19C—C20C	122.01 (18)
C5B—C4B—H4B	106.9	C18C—C19C—H19C	119.0
C1B—C5B—C4B	107.35 (14)	C20C—C19C—H19C	119.0
C1B—C5B—H5BA	110.2	C21C—C20C—C19C	119.72 (19)
C4B—C5B—H5BA	110.2	C21C—C20C—H20C	120.1
C1B—C5B—H5BB	110.2	C19C—C20C—H20C	120.1
C4B—C5B—H5BB	110.2	C20C—C21C—C22C	120.40 (19)
H5BA—C5B—H5BB	108.5	C20C—C21C—H21C	119.8
C11B—C6B—C7B	118.68 (19)	C22C—C21C—H21C	119.8
C11B—C6B—C1B	119.53 (18)	C21C—C22C—C23C	119.33 (18)
C7B—C6B—C1B	121.76 (18)	C21C—C22C—H22C	120.3
C8B—C7B—C6B	120.4 (2)	C23C—C22C—H22C	120.3
C8B—C7B—H7B	119.8	O3C—C23C—C22C	123.31 (17)
C6B—C7B—H7B	119.8	O3C—C23C—C18C	115.13 (16)
C9B—C8B—C7B	120.2 (2)	C22C—C23C—C18C	121.55 (18)
C9B—C8B—H8B	119.9	C23C—O3C—C24C	117.90 (15)
C7B—C8B—H8B	119.9	O3C—C24C—H24G	109.5
C8B—C9B—C10B	119.8 (2)	O3C—C24C—H24H	109.5
C8B—C9B—H9B	120.1	H24G—C24C—H24H	109.5
C10B—C9B—H9B	120.1	O3C—C24C—H24I	109.5
C9B—C10B—C11B	120.5 (2)	H24G—C24C—H24I	109.5
C9B—C10B—H10B	119.7	H24H—C24C—H24I	109.5
C11B—C10B—H10B	119.7		
			
O1A—C1A—C2A—O2A	−43.86 (18)	C1B—C6B—C11B—C10B	−177.79 (18)
C6A—C1A—C2A—O2A	−164.24 (14)	O2B—C2B—C12B—C17B	11.0 (2)
C5A—C1A—C2A—O2A	73.19 (17)	C3B—C2B—C12B—C17B	136.46 (19)
O1A—C1A—C2A—C12A	78.81 (19)	C1B—C2B—C12B—C17B	−105.0 (2)
C6A—C1A—C2A—C12A	−41.6 (2)	O2B—C2B—C12B—C13B	−167.48 (17)
C5A—C1A—C2A—C12A	−164.14 (16)	C3B—C2B—C12B—C13B	−42.0 (2)
O1A—C1A—C2A—C3A	−155.21 (15)	C1B—C2B—C12B—C13B	76.6 (2)
C6A—C1A—C2A—C3A	84.41 (17)	C17B—C12B—C13B—C14B	0.3 (3)
C5A—C1A—C2A—C3A	−38.16 (18)	C2B—C12B—C13B—C14B	178.85 (19)
O2A—C2A—C3A—C4A	−67.63 (18)	C12B—C13B—C14B—C15B	−0.9 (3)
C12A—C2A—C3A—C4A	170.73 (16)	C13B—C14B—C15B—C16B	0.7 (4)
C1A—C2A—C3A—C4A	44.18 (19)	C14B—C15B—C16B—C17B	0.1 (4)
C2A—C3A—C4A—C18A	−158.98 (17)	C15B—C16B—C17B—C12B	−0.6 (3)
C2A—C3A—C4A—C5A	−32.0 (2)	C13B—C12B—C17B—C16B	0.4 (3)
O1A—C1A—C5A—C4A	132.32 (17)	C2B—C12B—C17B—C16B	−178.11 (19)
C6A—C1A—C5A—C4A	−102.40 (19)	C3B—C4B—C18B—C19B	1.4 (3)
C2A—C1A—C5A—C4A	18.8 (2)	C5B—C4B—C18B—C19B	−121.0 (2)
C18A—C4A—C5A—C1A	134.97 (19)	C3B—C4B—C18B—C23B	−175.52 (17)
C3A—C4A—C5A—C1A	7.5 (2)	C5B—C4B—C18B—C23B	62.1 (2)
O1A—C1A—C6A—C7A	153.34 (17)	C23B—C18B—C19B—C20B	0.6 (3)
C5A—C1A—C6A—C7A	27.4 (2)	C4B—C18B—C19B—C20B	−176.4 (2)
C2A—C1A—C6A—C7A	−88.4 (2)	C18B—C19B—C20B—C21B	−1.4 (4)
O1A—C1A—C6A—C11A	−27.9 (2)	C19B—C20B—C21B—C22B	0.4 (4)
C5A—C1A—C6A—C11A	−153.85 (17)	C20B—C21B—C22B—C23B	1.3 (4)
C2A—C1A—C6A—C11A	90.3 (2)	C21B—C22B—C23B—O3B	176.2 (2)
C11A—C6A—C7A—C8A	0.8 (3)	C21B—C22B—C23B—C18B	−2.1 (3)
C1A—C6A—C7A—C8A	179.5 (2)	C19B—C18B—C23B—O3B	−177.27 (18)
C6A—C7A—C8A—C9A	0.9 (4)	C4B—C18B—C23B—O3B	−0.1 (3)
C7A—C8A—C9A—C10A	−1.1 (4)	C19B—C18B—C23B—C22B	1.1 (3)
C8A—C9A—C10A—C11A	−0.3 (4)	C4B—C18B—C23B—C22B	178.27 (19)
C9A—C10A—C11A—C6A	2.0 (3)	C22B—C23B—O3B—C24B	−3.6 (3)
C7A—C6A—C11A—C10A	−2.2 (3)	C18B—C23B—O3B—C24B	174.72 (19)
C1A—C6A—C11A—C10A	179.01 (18)	O1C—C1C—C2C—O2C	44.87 (18)
O2A—C2A—C12A—C13A	29.2 (2)	C6C—C1C—C2C—O2C	165.05 (14)
C3A—C2A—C12A—C13A	148.90 (18)	C5C—C1C—C2C—O2C	−72.76 (16)
C1A—C2A—C12A—C13A	−91.9 (2)	O1C—C1C—C2C—C3C	156.57 (14)
O2A—C2A—C12A—C17A	−148.79 (17)	C6C—C1C—C2C—C3C	−83.25 (17)
C3A—C2A—C12A—C17A	−29.1 (3)	C5C—C1C—C2C—C3C	38.94 (16)
C1A—C2A—C12A—C17A	90.1 (2)	O1C—C1C—C2C—C12C	−76.92 (18)
C17A—C12A—C13A—C14A	0.7 (3)	C6C—C1C—C2C—C12C	43.3 (2)
C2A—C12A—C13A—C14A	−177.40 (18)	C5C—C1C—C2C—C12C	165.45 (15)
C12A—C13A—C14A—C15A	0.9 (3)	O2C—C2C—C3C—C4C	69.39 (17)
C13A—C14A—C15A—C16A	−1.7 (3)	C12C—C2C—C3C—C4C	−168.72 (15)
C14A—C15A—C16A—C17A	0.8 (3)	C1C—C2C—C3C—C4C	−41.95 (17)
C15A—C16A—C17A—C12A	0.8 (3)	C2C—C3C—C4C—C18C	155.40 (15)
C13A—C12A—C17A—C16A	−1.6 (3)	C2C—C3C—C4C—C5C	28.12 (18)
C2A—C12A—C17A—C16A	176.49 (18)	O1C—C1C—C5C—C4C	−134.59 (15)
C3A—C4A—C18A—C23A	−168.32 (19)	C6C—C1C—C5C—C4C	99.72 (17)
C5A—C4A—C18A—C23A	70.4 (3)	C2C—C1C—C5C—C4C	−21.77 (17)
C3A—C4A—C18A—C19A	10.8 (3)	C18C—C4C—C5C—C1C	−130.87 (16)
C5A—C4A—C18A—C19A	−110.5 (2)	C3C—C4C—C5C—C1C	−3.16 (18)
C23A—C18A—C19A—C20A	−0.1 (3)	O1C—C1C—C6C—C11C	25.1 (2)
C4A—C18A—C19A—C20A	−179.2 (2)	C5C—C1C—C6C—C11C	151.88 (17)
C18A—C19A—C20A—C21A	1.1 (4)	C2C—C1C—C6C—C11C	−92.3 (2)
C19A—C20A—C21A—C22A	−0.3 (4)	O1C—C1C—C6C—C7C	−156.75 (16)
C20A—C21A—C22A—C23A	−1.4 (4)	C5C—C1C—C6C—C7C	−30.0 (2)
C21A—C22A—C23A—O3A	−178.4 (2)	C2C—C1C—C6C—C7C	85.8 (2)
C21A—C22A—C23A—C18A	2.5 (3)	C11C—C6C—C7C—C8C	0.9 (3)
C19A—C18A—C23A—O3A	179.10 (19)	C1C—C6C—C7C—C8C	−177.29 (16)
C4A—C18A—C23A—O3A	−1.7 (3)	C6C—C7C—C8C—C9C	0.1 (3)
C19A—C18A—C23A—C22A	−1.7 (3)	C7C—C8C—C9C—C10C	−0.8 (3)
C4A—C18A—C23A—C22A	177.4 (2)	C8C—C9C—C10C—C11C	0.6 (3)
C22A—C23A—O3A—C24A	8.7 (3)	C9C—C10C—C11C—C6C	0.4 (3)
C18A—C23A—O3A—C24A	−172.2 (2)	C7C—C6C—C11C—C10C	−1.1 (3)
O1B—C1B—C2B—O2B	−46.09 (18)	C1C—C6C—C11C—C10C	177.08 (18)
C6B—C1B—C2B—O2B	−164.67 (15)	O2C—C2C—C12C—C17C	−18.7 (2)
C5B—C1B—C2B—O2B	72.53 (16)	C3C—C2C—C12C—C17C	−139.00 (18)
O1B—C1B—C2B—C12B	75.0 (2)	C1C—C2C—C12C—C17C	101.2 (2)
C6B—C1B—C2B—C12B	−43.6 (2)	O2C—C2C—C12C—C13C	160.22 (17)
C5B—C1B—C2B—C12B	−166.40 (15)	C3C—C2C—C12C—C13C	39.9 (2)
O1B—C1B—C2B—C3B	−159.84 (14)	C1C—C2C—C12C—C13C	−79.8 (2)
C6B—C1B—C2B—C3B	81.58 (18)	C17C—C12C—C13C—C14C	0.5 (3)
C5B—C1B—C2B—C3B	−41.22 (16)	C2C—C12C—C13C—C14C	−178.47 (19)
O2B—C2B—C3B—C4B	−63.58 (18)	C12C—C13C—C14C—C15C	0.0 (3)
C12B—C2B—C3B—C4B	170.51 (15)	C13C—C14C—C15C—C16C	−0.4 (3)
C1B—C2B—C3B—C4B	43.51 (17)	C14C—C15C—C16C—C17C	0.3 (3)
C2B—C3B—C4B—C18B	−156.71 (15)	C15C—C16C—C17C—C12C	0.2 (3)
C2B—C3B—C4B—C5B	−28.22 (18)	C13C—C12C—C17C—C16C	−0.6 (3)
O1B—C1B—C5B—C4B	141.76 (15)	C2C—C12C—C17C—C16C	178.34 (19)
C6B—C1B—C5B—C4B	−97.88 (18)	C3C—C4C—C18C—C19C	0.2 (3)
C2B—C1B—C5B—C4B	24.37 (18)	C5C—C4C—C18C—C19C	122.14 (19)
C18B—C4B—C5B—C1B	129.23 (17)	C3C—C4C—C18C—C23C	176.88 (17)
C3B—C4B—C5B—C1B	1.50 (19)	C5C—C4C—C18C—C23C	−61.2 (2)
O1B—C1B—C6B—C11B	−34.6 (2)	C23C—C18C—C19C—C20C	0.5 (3)
C5B—C1B—C6B—C11B	−158.06 (17)	C4C—C18C—C19C—C20C	177.27 (18)
C2B—C1B—C6B—C11B	86.1 (2)	C18C—C19C—C20C—C21C	0.5 (3)
O1B—C1B—C6B—C7B	147.27 (17)	C19C—C20C—C21C—C22C	−0.6 (3)
C5B—C1B—C6B—C7B	23.8 (2)	C20C—C21C—C22C—C23C	−0.4 (3)
C2B—C1B—C6B—C7B	−92.0 (2)	C21C—C22C—C23C—O3C	−177.47 (19)
C11B—C6B—C7B—C8B	0.0 (3)	C21C—C22C—C23C—C18C	1.5 (3)
C1B—C6B—C7B—C8B	178.12 (18)	C19C—C18C—C23C—O3C	177.48 (17)
C6B—C7B—C8B—C9B	−0.1 (3)	C4C—C18C—C23C—O3C	0.6 (3)
C7B—C8B—C9B—C10B	−0.1 (3)	C19C—C18C—C23C—C22C	−1.6 (3)
C8B—C9B—C10B—C11B	0.5 (4)	C4C—C18C—C23C—C22C	−178.46 (17)
C9B—C10B—C11B—C6B	−0.6 (3)	C22C—C23C—O3C—C24C	−5.8 (3)
C7B—C6B—C11B—C10B	0.4 (3)	C18C—C23C—O3C—C24C	175.19 (18)

### (1R,2S,4r)-4-(2-Methoxyphenyl)-1,2-diphenylcyclopentane-1,2-diol (II) Hydrogen-bond geometry (Å, º)

**Table:**

*D*—H···*A*	*D*—H	H···*A*	*D*···*A*	*D*—H···*A*
O1*A*—H1*A*···O2*C*^i^	0.85 (3)	2.08 (3)	2.8931 (19)	160 (3)
O2*A*—H2*A*···O1*A*	0.88 (3)	2.04 (3)	2.605 (2)	121 (2)
O1*B*—H1*B*···O2*B*	0.90 (3)	2.05 (3)	2.590 (2)	117 (2)
O2*B*—H2*B*···O2*A*	0.83 (2)	1.98 (2)	2.802 (2)	170 (2)
O1*C*—H1*C*···O1*B*	0.88 (3)	1.96 (3)	2.833 (2)	171 (2)
O2*C*—H2*C*···O1*C*	0.85 (3)	2.00 (3)	2.587 (2)	125 (2)

Symmetry code: (i) *x*−1, *y*−1, *z*.

## References

[bb1] Bruker (2018). *APEX3* and *SAINT*. Bruker AXS Inc., Madison, Wisconsin, USA.

[bb2] Chen, H.-Y., Mialon, L., Abboud, K. A. & Miller, S. A. (2012). *Organometallics*, **31**, 5252–5261.

[bb3] Choi, T., Cizmeciyan, D., Khan, S. I. & Garcia-Garibay, M. A. (1995). *J. Am. Chem. Soc.* **117**, 12893–12894.

[bb4] Davies, J. E., Mays, M. J., Raithby, P. R., Sarveswaran, K. & Solan, G. A. (2000). *Chem. Commun.* pp. 1313–1314.

[bb5] Deck, P. A., McCauley, B. D. & Slebodnick, C. (2006). *J. Organomet. Chem.* **691**, 1973–1983.

[bb6] Groom, C. R., Bruno, I. J., Lightfoot, M. P. & Ward, S. C. (2016). *Acta Cryst.* B**72**, 171–179. 10.1107/S2052520616003954 PMC4822653 27048719

[bb7] Hirsch, S. S. & Bailey, W. J. (1978). *J. Org. Chem.* **43**, 4090–4094.

[bb8] Krause, L., Herbst-Irmer, R., Sheldrick, G. M. & Stalke, D. (2015). *J. Appl. Cryst.* **48**, 3–10. 10.1107/S1600576714022985 PMC4453166 26089746

[bb9] Macrae, C. F., Edgington, P. R., McCabe, P., Pidcock, E., Shields, G. P., Taylor, R., Towler, M. & van de Streek, J. (2006). *J. Appl. Cryst.* **39**, 453–457.

[bb10] Minyaev, M. E., Nifant’ev, I. E., Shlyakhtin, A. V., Ivchenko, P. V. & Lyssenko, K. A. (2018). *Acta Cryst.* C**74**, 548–557. 10.1107/S2053229618005090 29726463

[bb11] Minyaev, M. E., Roitershtein, D. M., Nifant’ev, I. E., Ananyev, I. V., Minyaeva, T. V. & Mikhaylyev, T. A. (2015). *Acta Cryst.* C**71**, 491–498. 10.1107/S2053229615009857 26044332

[bb12] Minyaev, M. E., Vinogradov, A. A., Roitershtein, D. M., Borisov, R. S., Ananyev, I. V., Churakov, A. V. & Nifant’ev, I. E. (2016). *J. Organomet. Chem.* **818**, 128–136.

[bb13] Nifant’ev, I. E., Shlyakhtin, A. V., Bagrov, V. V., Minyaev, M. E., Churakov, A. V., Karchevsky, S. G., Birin, K. P. & Ivchenko, P. V. (2017). *Dalton Trans.* **46**, 12132–12146. 10.1039/c7dt02469j 28869269

[bb14] Nifant’ev, I. E., Shlyakhtin, A. V., Tavtorkin, A. N., Ivchenko, P. V., Borisov, R. S. & Churakov, A. V. (2016). *Catal. Commun.* **87**, 106–111.

[bb15] Roitershtein, D. M., Minyaev, M. E., Mikhaylyuk, A. A., Lyssenko, K. A., Glukhov, I. V. & Belyakov, P. A. (2012). *Russ. Chem. Bull.* **61**, 1726–1732.

[bb16] Roitershtein, D. M., Puntus, L. N., Vinogradov, A. A., Lyssenko, K. A., Minyaev, M. E., Dobrokhodov, M. D., Taidakov, I. V., Varaksina, E. A., Churakov, A. V. & Nifant’ev, I. E. (2018). *Inorg. Chem.* **57**, 10199–10213. 10.1021/acs.inorgchem.8b01405 30051707

[bb17] Sheldrick, G. M. (2008). *Acta Cryst.* A**64**, 112–122. 10.1107/S0108767307043930 18156677

[bb18] Sheldrick, G. M. (2015). *Acta Cryst.* C**71**, 3–8.

[bb19] Thornberry, M. P., Reynolds, N. T., Deck, P. A., Fronczek, F. R., Rheingold, A. L. & Liable-Sands, L. M. (2004). *Organometallics*, **23**, 1333–1339.

[bb20] Thornberry, M. P., Slebodnick, C., Deck, P. A. & Fronczek, F. R. (2000). *Organometallics*, **19**, 5352–5369.

[bb21] Visseaux, M., Zinck, P., Terrier, M., Mortreux, A. & Roussel, P. (2008). *J. Alloys Compd.* **451**, 352–357.

[bb22] Westrip, S. P. (2010). *J. Appl. Cryst.* **43**, 920–925.

[bb23] Wu, Q.-L., Su, Q., Ye, L. & Mu, Y. (2007). *Acta Cryst.* E**63**, m1160–m1161.

[bb24] Xu, J., Gao, W., Zhang, Y., Li, J. & Mu, Y. (2007). *J. Organomet. Chem.* **692**, 1505–1510.

[bb25] Xu, J., Mu, X., Zhang, Y., Su, Q., Ni, J. & Mu, Y. (2006). *J. Chem. Res. (S)*, pp. 552–554.

[bb26] Yang, L., Ye, J., Xu, L., Yang, X., Gong, W., Lin, Y. & Ning, G. (2012). *RSC Adv.* **2**, 11529–11535.

[bb27] Ye, J., Gao, Y., He, L., Tan, T., Chen, W., Liu, Y., Wang, Y. & Ning, G. (2016). *Dyes Pigments*, **124**, 145–155.

[bb28] Ye, J., Huang, X., Li, Y., Zheng, T., Ning, G., Liang, J., Liu, Y. & Wang, Y. (2017). *Dyes Pigments*, **147**, 465–475.

[bb29] Zhang, F., Mu, Y., Wang, J., Shi, Z., Bu, W., Hu, S., Zhang, Y. & Feng, S. (2000). *Polyhedron*, **19**, 1941–1947.

[bb30] Zhang, X., Ye, J., Xu, L., Yang, L., Deng, D. & Ning, G. (2013). *J. Lumin.* **139**, 28–34.

[bb31] Zhang, Y., Wang, J., Mu, Y., Shi, Z., Lü, C., Zhang, Y., Qiao, L. & Feng, S. (2003). *Organometallics*, **22**, 3877–3883.

